# Plasma membrane architecture protects *Candida albicans* from killing by copper

**DOI:** 10.1371/journal.pgen.1007911

**Published:** 2019-01-11

**Authors:** Lois M. Douglas, James B. Konopka

**Affiliations:** Department of Molecular Genetics and Microbiology, Stony Brook University, Stony Brook, New York, United States of America; Johns Hopkins University Bloomberg School of Public Health, UNITED STATES

## Abstract

The ability to resist copper toxicity is important for microbial pathogens to survive attack by innate immune cells. A *sur7*Δ mutant of the fungal pathogen *Candida albicans* exhibits decreased virulence that correlates with increased sensitivity to copper, as well as defects in other stress responses and morphogenesis. Previous studies indicated that copper kills *sur7Δ* cells by a mechanism distinct from the known resistance pathways involving the Crp1 copper exporter or the Cup1 metallothionein. Since Sur7 resides in punctate plasma membrane domains known as MCC/eisosomes, we examined overexpression of *SUR7* and found that it rescued the copper sensitivity of a mutant that fails to form MCC/eisosomes (*pil1*Δ *lsp1*Δ), indicating that these domains act to facilitate Sur7 function. Genetic screening identified new copper-sensitive mutants, the strongest of which were similar to *sur7*Δ in having altered plasma membranes due to defects in membrane trafficking, cortical actin, and morphogenesis (*rvs161*Δ, *rvs167*Δ, and *arp2*Δ *arp3*Δ). Consistent with the mutants having altered plasma membrane organization, they were all more readily permeabilized by copper, which is known to bind phosphatidylserine and phosphatidylethanolamine and cause membrane damage. Although these phospholipids are normally localized to the intracellular leaflet of the plasma membrane, their exposure on the surface of the copper-sensitive mutants was indicated by increased susceptibility to membrane damaging agents that bind to these phospholipids. Increased copper sensitivity was also detected for a *drs2*Δ mutant, which lacks a phospholipid flippase that is involved in maintaining phospholipid asymmetry. Copper binds phosphatidylserine with very high affinity, and deleting *CHO1* to prevent phosphatidylserine synthesis rescued the copper sensitivity of *sur7*Δ cells, confirming a major role for phosphatidylserine in copper sensitivity. These results highlight how proper plasma membrane architecture protects fungal pathogens from copper and attack by the immune system, thereby opening up new avenues for therapeutic intervention.

## Author summary

The transition metal copper is used by the innate immune system to attack microbial pathogens. To better understand how the human fungal pathogen *Candida albicans* resists this type of stress, we screened for mutants that were more susceptible to killing by copper. Interestingly, we identified a new class of copper-sensitive mutants whose plasma membranes are more readily permeabilized by copper. The common characteristic of these new copper-sensitive mutants is that they have an altered cell surface, which weakened their resistance to copper. These results help to explain the toxic effects of copper and suggest novel therapeutic strategies for fungal infections.

## Introduction

The human fungal pathogen *C*. *albicans* typically grows as a commensal organism on human mucosa. However, *C*. *albicans* can cause severe mucosal infections or lethal systemic infections when the immune system is impaired [1, 2]. Serious infections also occur when conditions promote an overgrowth of *C*. *albicans* that overwhelms the immune system. This can happen as a consequence of the use of antibacterial antibiotics that disrupt the microbiota or as the result of biofilm formation on medical devices and catheters. In order to survive in a human host, *C*. *albicans* must be able to resist a wide range of stressful conditions promoted by the immune system. This includes elevated temperature, antimicrobial peptides, oxidation, and nitrosylation [3–5]. Copper has recently been recognized as a form of stress encountered by microbes in vivo [6–8], and *C*. *albicans* cells have been reported to experience copper stress at sites of infection [9, 10]. For example, stimulation of macrophages with interferon-γ leads to increased expression of the copper importer *CTR1* and translocation of the ATP7A copper transporter to the phagosomal membrane where it pumps copper into the phagosome [11]. Copper can react with H_2_O_2_ produced by the oxidative burst in phagosomes to form a broader array of damaging reactive oxygen species [6, 12, 13].

Cells have a complex relationship with copper in that excess copper is toxic, but too little copper is also bad, as cells require this metal as an essential cofactor for many enzymes. The ability of copper to transition between cuprous (Cu^1+^) and cupric (Cu^2+^) oxidation states facilitates the catalysis of many important electron transfer reactions in the cell. Fungal cells therefore use several mechanisms to tightly regulate the levels of copper such that there appears to be less than one free copper molecule per cell [14, 15]. One mechanism is that excess copper inactivates the Mac1 transcription factor to turn off expression of the copper importer *CTR1*, thereby decreasing copper uptake [16]. Once inside the cell, intracellular copper is typically sequestered by a chaperone protein for delivery to an appropriate metalloprotein, such as the role of Ccs1 in delivering copper to the Sod1 superoxide dismutase [17]. Excess cytoplasmic copper is bound by scavenger proteins, such as the Cup1 and Crd2 metallothionein proteins in *C*. *albicans*, or stored in the vacuole [18–21]. In *Cryptococcus neoformans*, metallothioneins play a key role in copper resistance and are important for virulence [7, 22]. In *C*. *albicans*, the major role in resisting copper is carried out by Crp1, a plasma membrane exporter which pumps excess copper out of the cell, while metallothioneins play a less important role [18, 19]. Consistent with this, *CRP1* is important for virulence of *C*. *albicans* [9]. The *C*. *albicans* copper resistance genes *CUP1* and *CRP1* are induced in response to excess copper by the Cup2 transcription factor [18, 19, 23, 24], and copper resistance also appears to be regulated by the PKA pathway [25].

Recent studies revealed that the Sur7 plasma membrane protein promotes resistance of *C*. *albicans* to copper by a mechanism that is distinct from the known pathways mediated by Crp1 and Cup1 [26]. Sur7 is a tetraspan integral membrane protein that localizes to punctate patches in the plasma membrane known as MCC domains, each of which is associated with a complex of cytoplasmic proteins known as an eisosome [27–29]. These domains correspond to ~250 nm long furrows in the plasma membrane created by the Pil1 and Lsp1 proteins [30–32]. The *sur7Δ* mutant showed strong defects in virulence and a decreased ability to grow in macrophages that correlated with increased sensitivity to copper [26]. The *sur7Δ* mutant has additional defects in morphogenesis, plasma membrane, and cell wall composition that likely contribute to the virulence defects [31, 33–35].

Many of the toxic effects of copper are attributed to its redox properties, which can lead to oxidation of protein, lipids and DNA [6–8, 36]. Excess copper can also inhibit growth of microbes by having direct toxic effects on a wide range of macromolecules, especially those containing thiols, such as the iron sulfur cluster proteins [6, 7, 37]. Previous studies showed that copper can permeabilize the plasma membranes of diverse cell types, including bacterial, fungal, plant, and animal [12, 36, 38–41]; however, the mechanism is not understood. To better define the novel process by which Sur7 promotes resistance to copper, we used genetic approaches to screen for *C*. *albicans* mutants that are more susceptible to killing by copper. The strongest mutants identified are similar to *sur7Δ* cells in having defects in membrane trafficking and cortical actin organization that cause broad changes in plasma membrane architecture (*pil1Δ lsp1Δ*, *rvs161Δ*, *rvs167Δ*, and *arp2Δ arp3Δ)*. Interestingly, these mutants were all permeabilized more readily by copper. Analysis of cell surface lipids implicated exposure of phosphatidylserine (PS) in copper sensitivity, which correlates with studies on model membranes demonstrating that copper binds PS with high affinity and promotes membrane damage [42–44]. These results provide important new insight into how plasma membrane architecture is organized to protect the cell surface from attack by copper. These results also have significance for the development of novel therapeutic approaches and for the effective use of metallic copper surfaces as an antimicrobial strategy [45, 46].

## Results

### MCC/eisosomes facilitate the role of Sur7 in resistance to copper

**Fig 1:**
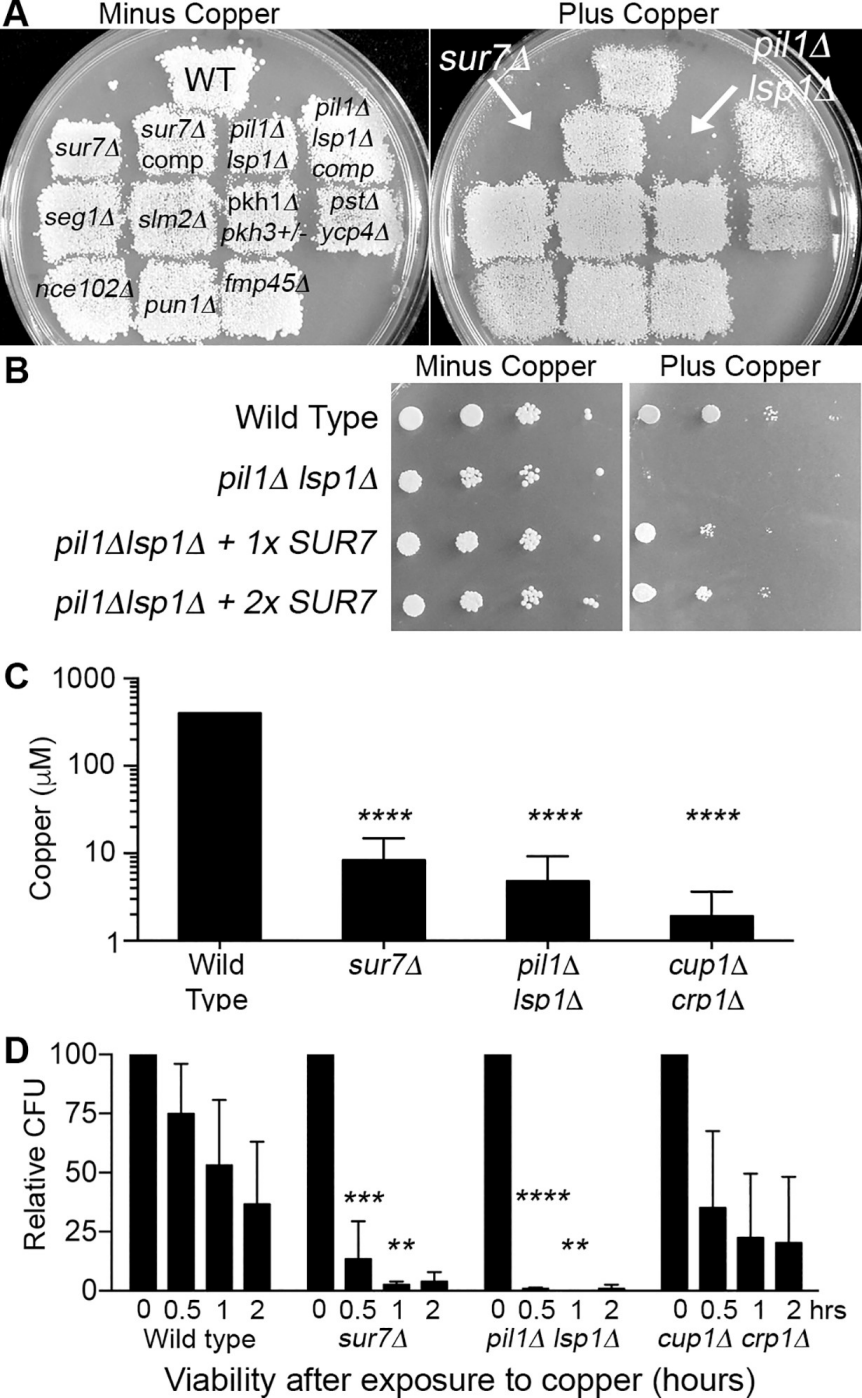
MCC/eisosome proteins Sur7, Pil1, and Lsp1 are important for resistance of *Candida albicans* to copper. (A) *C*. *albicans* strains lacking the indicated MCC/eisosome genes were replica-plated onto minimal medium containing either 0 μM or 500 μM CuSO_4_ and incubated at 30°C for 48 hr. Only the *sur7Δ* and the *pil1Δ lsp1Δ* double deletion demonstrated strong sensitivity. (B) *SUR7* overexpression partially rescued the copper sensitivity of the *pil1Δ lsp1Δ* strain. *pil1Δ lsp1Δ* strains containing zero, one, or two extra copies of the *SUR7* gene were spotted onto minimal medium in the absence or presence of 1 mM CuSO_4_ and incubated for 48 hr at 30°C. (C) The highest concentration of copper at which the indicated strains could grow after incubation in synthetic medium in 96-well plates for 48 hr at 37°C with different concentrations of copper. The *sur7Δ*, *pil1Δ lsp1Δ*, and known copper sensitive mutant *cup1Δ crp1Δ* could only grow at lower concentrations of copper than the wild-type strain, indicating increased sensitivity. (D) Time course study of viability after exposure to copper. Cells were incubated in H_2_O or 0.5 μM CuSO_4_ at 37°C for the indicated time and then an aliquot was spread on YPD medium to determine viable colony forming units (CFU). All deletion mutants exhibited lower viability after incubation in CuSO_4_. Images in panel A are representative of three independent experiments, and those in panel B of two independent experiments, all performed on different days. The results in panel C represent averages of three independent experiments and those in panel D of four independent experiments, all performed on different days. Error bars indicate SD. ****, P<0.001. ***, P<0.01. **, P<0.01 by two-way ANOVA. Strains used were: WT, wild-type DIC185; *sur7Δ* (YJA11); *sur7Δ* comp. (YJA12); *pil1Δ lsp1Δ* (YHXW21-1); *pil1Δ lsp1Δ* comp (+ *LSP1*, YHXW23-1); *seg1Δ* (YLD182-9-10-2); *slm2Δ* (YHXW36-1); *pkh1Δ pkh3Δ/PKH3* (YLD146-11-2-5); *pst1Δ pst2Δ pst3Δ ycp4Δ* (LLF060); *nce102Δ* (YHXW14); *pun1Δ* (YLD204-2); *fmp45Δ* (YHXW3), *pil1Δ lsp1Δ* + 1x*SUR7* (YHXW45-1), *pil1Δ lsp1Δ* + 2x*SUR7* (YHXW46-1), and *cup1Δ crp1Δ* (KC25).

To determine whether other proteins present in the MCC/eisosome domains of the *C*. *albicans* plasma membrane contribute to copper resistance, we tested 8 different mutant strains for their ability to grow on agar medium containing 500 μM CuSO_4_ (Fig 1A). All of the mutants exhibited wild-type growth, except the *pil1Δ lsp1Δ* mutant, which was similar to the *sur7Δ* strain in failing to show detectable growth. Pil1 and Lsp1 are membrane binding BAR domain proteins that function to form the eisosome domains [27, 30, 32]. MCC/eisosome domains facilitate Sur7 function by promoting its stability and localization in the plasma membrane [31]. We therefore tested the effects of overexpressing *SUR7* in the *pil1Δ lsp1Δ* mutant and found that it strongly rescued the growth of *pil1Δ lsp1Δ* cells on copper-containing medium (Fig 1B), similar to the way that overexpression of *SUR7* was found previously to rescue many of the cell wall and morphogenesis mutant phenotypes of *pil1Δ lsp1Δ* cells [31]. These results indicate that Sur7 plays the key role in copper resistance, and that Pil1 and Lsp1 act to facilitate this role.

To quantify the sensitivity to copper, mutant strains were grown in liquid cultures containing different concentrations of copper. Both the *sur7Δ* and the *pil1Δ lsp1Δ* mutants showed greatly increased sensitivity to copper, as they were inhibited by about a 100-fold lower dose of copper under these growth conditions (Fig 1C). Significantly, they were nearly as susceptible to copper as the highly sensitive *cup1Δ crp1Δ* mutant, which lacks both the metallothionein and the copper exporter genes [18]. The concentrations of copper needed to inhibit cell growth were affected by the growth medium, as the inclusion of components such as amino acids or yeast extract elevated the concentration of copper needed to kill cells, presumably because these media additives can chelate the copper. Therefore, these tests were done with minimal medium.

Time course studies were carried out by treating cells with 0.5 μM copper in water and then measuring the viable colony forming units (CFUs). Interestingly, whereas wild type cells slowly lost viability over a 2 hr incubation, the *sur7Δ* and *pil1Δ lsp1Δ* mutant cells rapidly lost viability as most of the cells were dead after 30 min (Fig 1D). The *cup1Δ crp1Δ* strain appeared to give intermediate sensitivity in this short-term assay, although variability in the CFU assays limited the statistical significance of these results. This was more readily observed in an independent set of time course assays performed under more acidic conditions that prolong the time course (S1 Fig).

### Genes that function in membrane trafficking and morphogenesis affect *C*. *albicans* copper resistance

**Fig 2:**
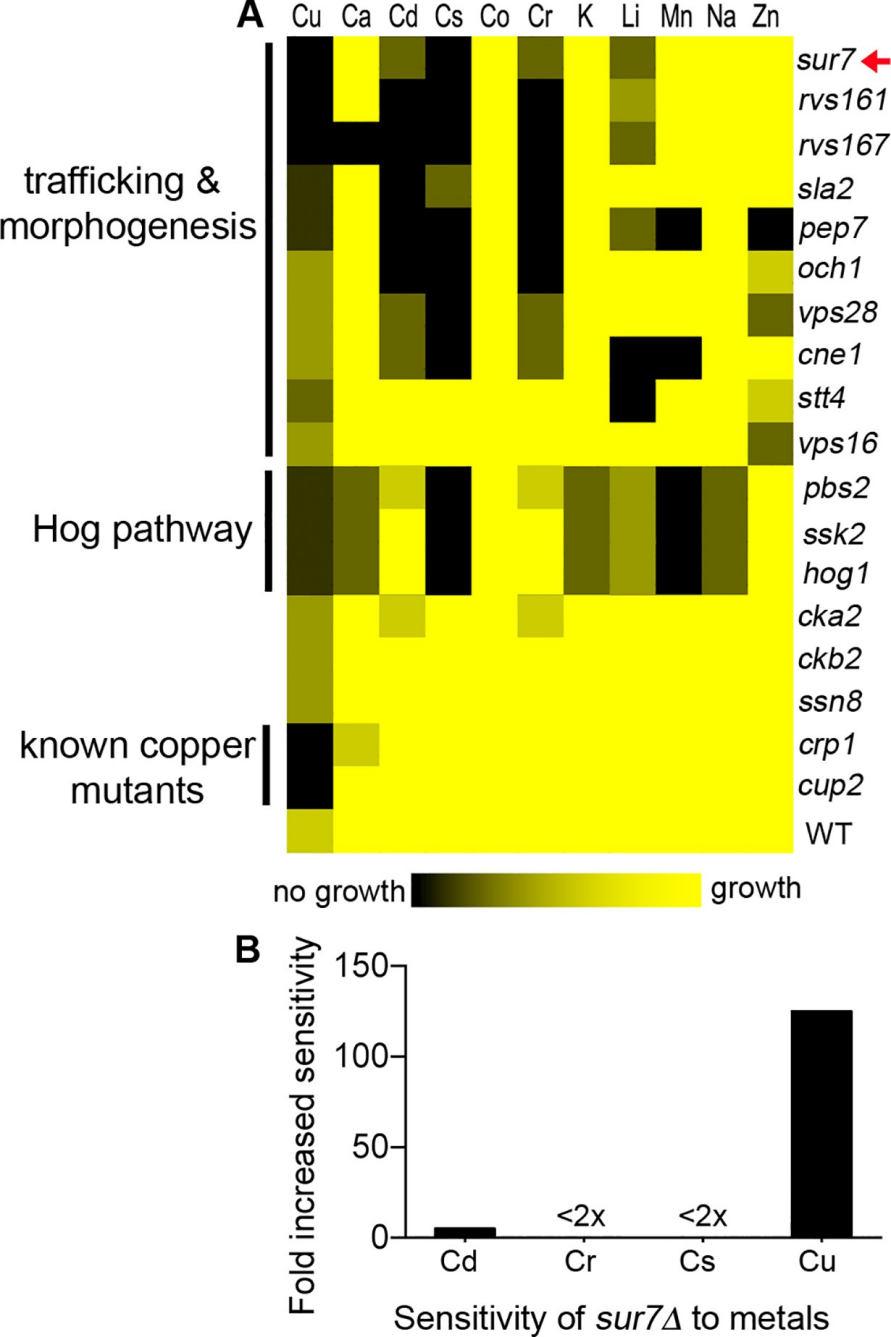
Genes that function in stress response, morphogenesis, and membrane trafficking influence copper resistance. (A) *C*. *albicans* strains were replica-plated onto minimal synthetic media containing copper or another indicated metal, and then the extent of growth was recorded and summarized in a heat map. The mutant strains clustered into three major groups. The *sur7Δ* mutant clustered with other mutants known to be defective in trafficking and morphogenesis, a separate cluster included the *HOG1* pathway mutants that are sensitive to stress, and the known copper mutants *crp1Δ* and cup2*Δ* clustered. The results represent a compilation of three experiments for each metal performed on different days. (B) The sensitivity of *sur7Δ* to cadmium (Cd), chromium, (Cr), and cesium (Cs) was further tested by incubating cells in different concentrations of these metals in synthetic medium a 96-well plate, since replica-plating indicated some degree of growth inhibition in response to these metals. Results indicated that *sur7Δ* cells only showed strong sensitivity to copper. The graph represents the averages of two to three independent experiments performed on different days.

To better define how cells resist copper toxicity, we screened libraries of *C*. *albicans* mutant strains [23, 47, 48], along with deletion strains in our own collection, by replica-plating cells onto copper-containing medium. The most sensitive mutants were also tested for growth in the presence of 10 other metals to examine the specificity of the copper phenotype. A heat map summary (Fig 2A) of the 18 most copper-sensitive mutants revealed that they mainly fell into three categories. One expected category included mutants lacking known copper resistance genes (*crp1Δ* and *cup2Δ*) that were characterized by high sensitivity to copper, but not the other metals. A second group contained HOG MAP kinase pathway mutants (*pbs2Δ*, *ssk2Δ*, *hog1Δ*), and the third was comprised of *sur7Δ* and other mutants with defects in morphogenesis and membrane trafficking (Fig 2A). These latter two mutant categories could be readily distinguished by their sensitivity to other metals. For example, the HOG pathway mutants were inhibited by calcium and manganese, whereas many of the morphogenesis mutants were sensitive to cadmium and chromium (Fig 2A). Although *sur7Δ* cells appeared more sensitive to cadmium, chromium, and cesium when assayed for growth by replica-plating onto agar plates, growth in liquid cultures containing different concentrations of metals indicated that the *sur7Δ* mutant was only weakly more susceptible to these other metals (Fig 2B). Thus, the *sur7Δ* mutant is preferentially affected by copper.

### Sensitivity to oxidative stress is not the chief cause of copper toxicity for *sur7Δ* cells

**Fig 3:**
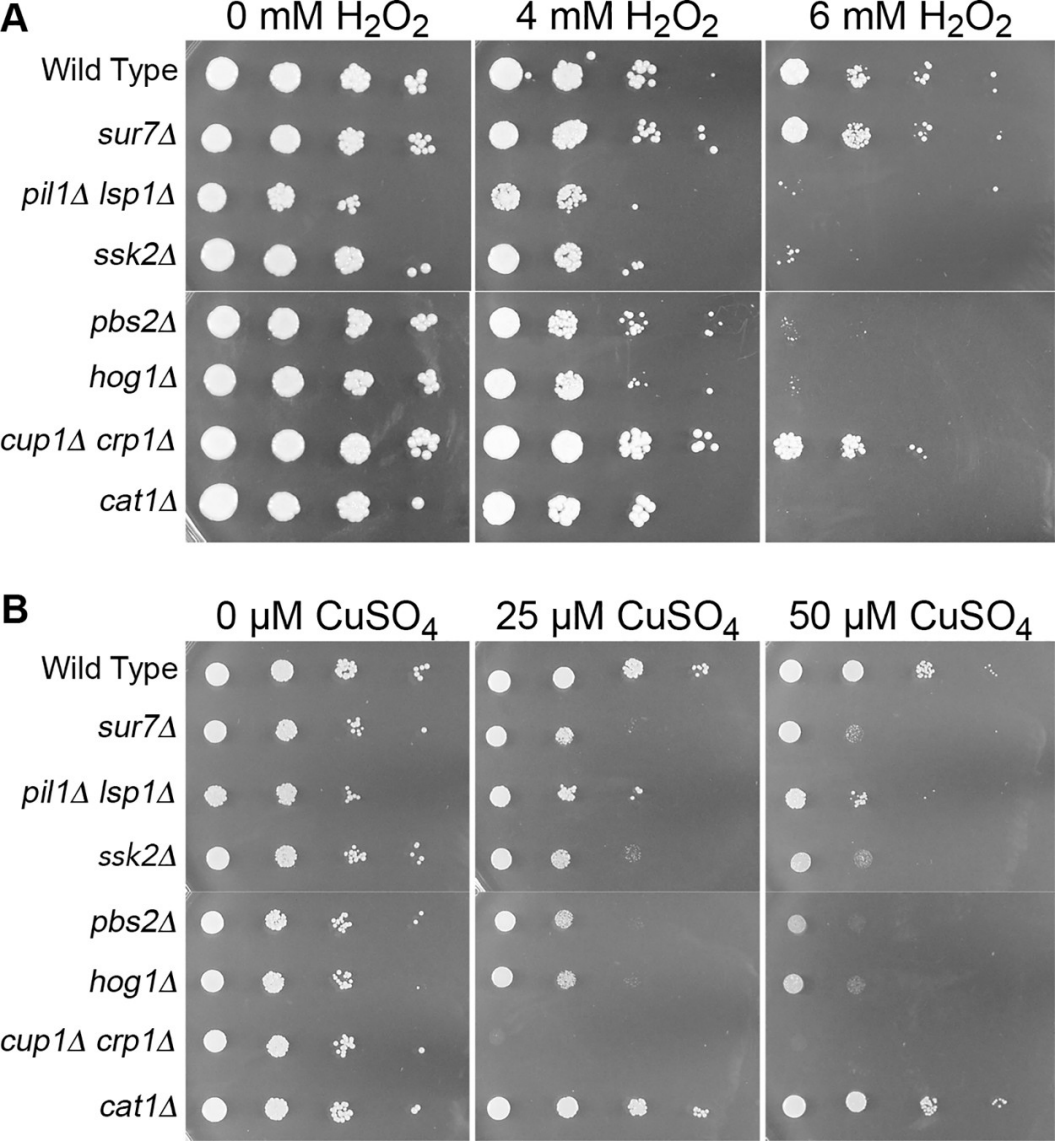
Copper sensitivity does not correlate with sensitivity to oxidative stress. Dilutions of the indicated cells were spotted onto (A) YPD containing H_2_O_2_ or (B) synthetic medium with CuSO_4_. A *cat1Δ* catalase deletion served as a control as it is highly sensitive to oxidative stress, but grew to wild type levels on CuSO_4_ media. Conversely, the known copper-sensitive mutant *cup1Δ crp1Δ* grew well on H_2_O_2_, but failed to grow on either CuSO_4_ concentration. Hog1 pathway strains *pbs2Δ*, *sskΔ 2*, and *hog1Δ* all displayed sensitivity to increasing concentrations of both H_2_O_2_ and CuSO_4_, as did *pil1Δ lsp1Δ*. *sur7Δ* was inhibited by copper but grew similar to wild type on H_2_O_2_ media. Images are representative of two independent experiments performed on different days. Strains used were wild type, DIC185, *sur7Δ* (YJA11), *pil1Δ lsp1Δ* (YHXW21-1), *ssk2Δ* (YLD185-7), *pbs2Δ* (YLD197-1), *hog1Δ* (YLD184-3), *cat1Δ* (MT505-A), and *cup1Δ crp1Δ* (KC25).

We next examined whether the copper sensitivity of *sur7Δ* and other *C*. *albicans* mutants identified in the screen was due to oxidative stress generated by copper. Copper is known to react with compounds such as H_2_O_2_ to generate diverse reactive oxygen species (ROS) that damage cells [6]. This is due in part to the ability of copper to transition between cuprous (Cu^1+^) and cupric (Cu^2+^) oxidation states, enabling it to undergo Fenton-like chemical reactions. Interestingly, the *sur7Δ* mutant showed at most a weak increase in sensitivity to oxidative stress when grown on medium containing H_2_O_2_ (Fig 3A). In contrast, control strains showed that a catalase mutant (*cat1Δ*) and the HOG pathway mutants (*pbs2Δ*, *ssk2Δ*,and *hog1Δ*) displayed increased sensitivity to H_2_O_2_, as expected based on previously published data [49, 50]. The *cat1Δ* catalase mutant grew well on medium containing copper, indicating that the presence of this metal in the medium did not cause high levels of oxidative stress (Fig 3B). In addition, the *cup1Δ crp1Δ* strain, which is very sensitive to copper, grew similar to the wild type control strain on medium containing H_2_O_2_. Interestingly, the *pil1Δ lsp1Δ* mutant was more sensitive to H_2_O_2_, which is likely to be due to effects on a family of four related antioxidant proteins that localize to eisosomes [51]. Altogether, these results indicate that the copper sensitivity of the *sur7Δ* mutant is not due to increased susceptibility to oxidative stress.

### Actin organization promotes resistance of *C*. *albicans* to copper

**Fig 4:**
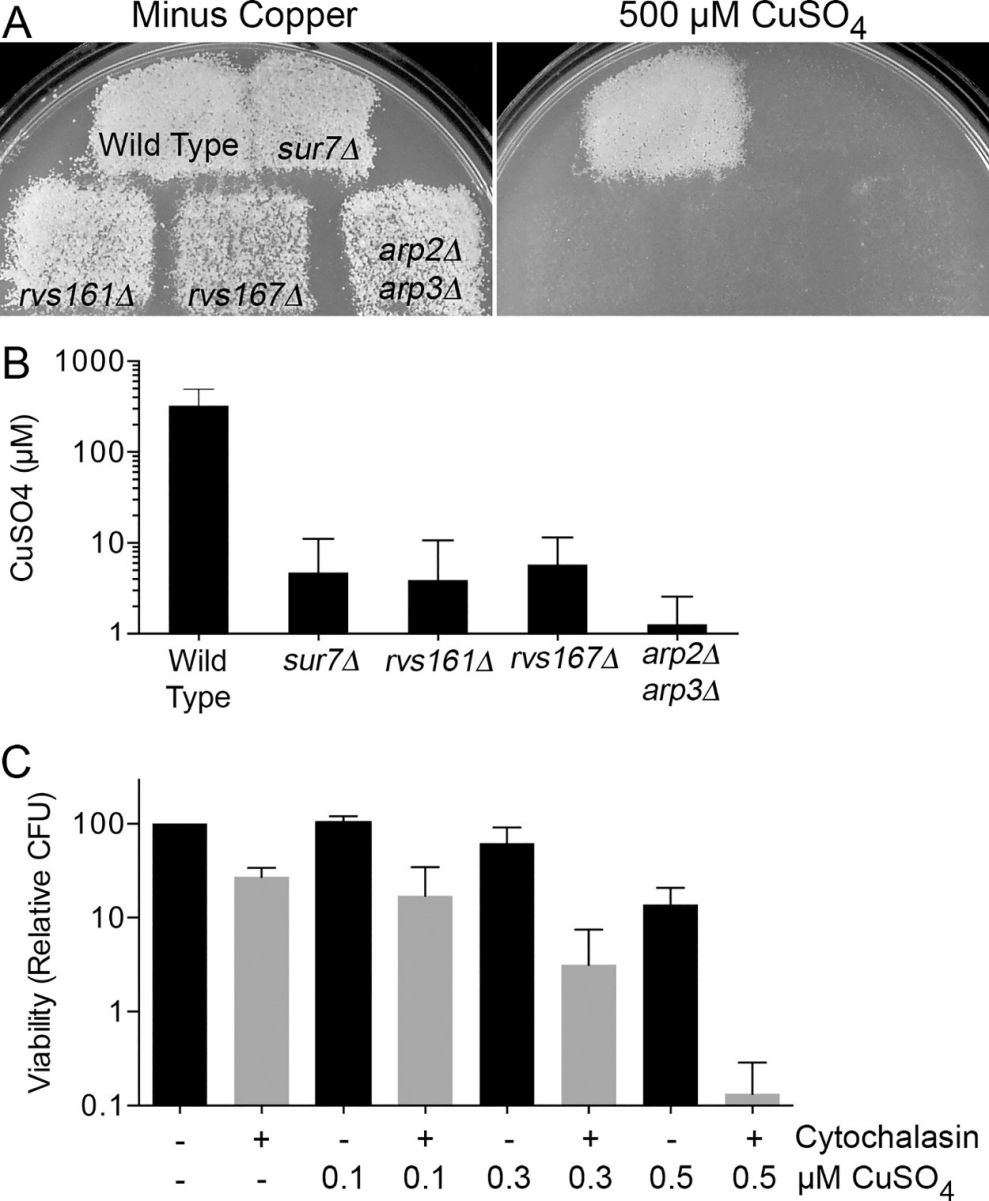
Actin defects contribute to copper sensitivity in *C*. *albicans*. (A) To further analyze the effects of copper on morphogenesis and trafficking mutants, *rvs161Δ*, *rvs167Δ*, and *arp2Δ arp3Δ* were replica-plated onto synthetic medium with 500 μM CuSO_4_. Only the wild type DIC185 was able to grow, indicating the extreme sensitivity of the deletion mutant strains. Images are representative of two independent experiments performed on different days. (B) Quantification of sensitivity by incubating mutant cells in a dilution series of copper concentrations in synthetic medium demonstrated that the *arp2Δ arp3Δ* mutant was the most sensitive, being inhibited by concentrations above 3.2 μM. The wild type, DIC185, was able to grow in 400 μM CuSO_4_. The graph represents the averages of four independent experiments performed on different days. (C) To determine whether an intact actin cytoskeleton is essential for resistance to copper, wild type DIC185 cells were treated with cytochalasin A in synthetic medium for 2 hr to depolarize the actin cytoskeleton, and then viability was assayed following incubation in the indicated concentration of CuSO_4_ for 2 hr. Relative colony-forming units (CFUs) decreased significantly with depolymerization of actin and exposure to increasing levels of copper. The graph represents averages of four independent experiments performed on different days. Strains used were DIC185, *sur7Δ* (YJA11), *rvs161Δ* (YLD14-3), *rvs167Δ* (YLD16), and *arp2Δ arp3Δ* (CaEE27).

Since the members of the largest group of copper-sensitive mutants we identified were similar to *sur7Δ* in having defects in membrane trafficking and morphogenesis, we examined three other mutants that are known to have strong defects in these processes and to also show altered plasma membrane organization. Two mutants, *rvs161Δ* and *rvs167Δ*, lack BAR domain proteins that are needed for proper cortical actin organization and play an important role in the scission phase of endocytosis [52]. In addition, we tested a double *arp2Δ arp3Δ* mutant of the actin-related proteins Arp2 and Arp3 that form a complex that is needed for cortical actin localization and efficient endocytosis [53]. All three strains showed increased inhibition of growth when replica-plated onto agar medium containing copper (Fig 4A). Incubation in a range of copper concentrations in liquid media showed that all three mutants were similar to *sur7Δ* in that they could only resist low concentrations of copper. The *arp2Δ arp3Δ* strain was the most sensitive, as it could only grow in copper concentrations up to 3.2 μM, while the wild type grew at ≥400 μM (Fig 4B).

A common feature of the copper-sensitive mutants *sur7Δ*, *pil1Δ lsp1Δ*, *rvs161Δ*, *rvs167Δ*, and *arp2Δ arp3Δ* is that they have abnormal actin organization [33, 52, 53]. We therefore examined the possibility that resistance to copper is dependent upon an intact actin cytoskeleton. This was tested by treating the wild type control strain DIC185 with the actin polymerization inhibitor cytochalasin A for 2 hr to disrupt the actin cytoskeleton, and then incubating the cells in the presence or absence of different concentrations of copper. Interestingly, over the range of copper concentrations used, cells incubated in water showed about a 5-fold decrease in viability, whereas cytochalasin A-treated cells showed about a 100-fold decrease in viability (Fig 4C). This synergistic effect on the loss of viability when cytochalasin A-treated cells were exposed to copper supports the conclusion that normal actin cytoskeleton and plasma membrane organization promote resistance of *C*. *albicans* to copper.

### Copper permeabilizes the plasma membrane of *sur7Δ* and other morphogenesis mutants

**Fig 5:**
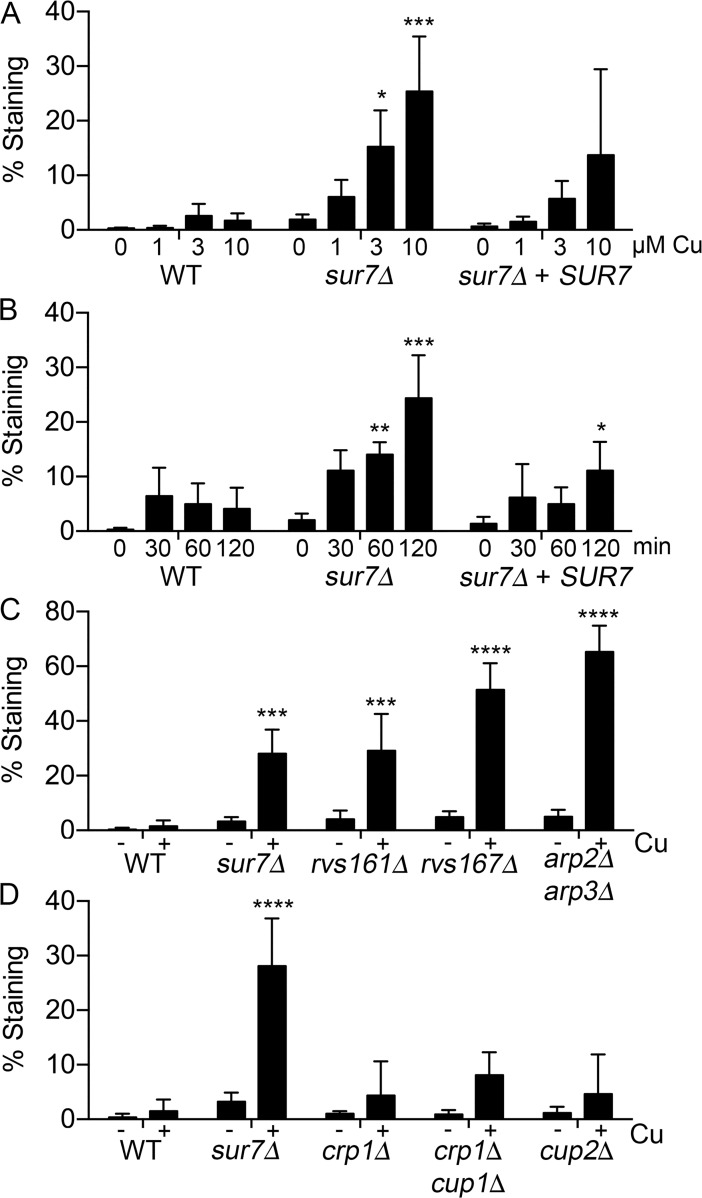
Copper permeabilizes plasma membranes of *C*. *albicans* copper-sensitive strains with morphogenesis defects. (A) Log phase *sur7Δ* cells were incubated in water containing the indicated concentrations of CuSO_4_ for 2 hr at 37°C and then stained with SYTOX Green to assess the integrity of the plasma membrane. The *sur7Δ* mutant displayed increased permeabilization of the plasma membrane with increased copper concentration compared to the wild type and *Sur7* complemented strains. (B) Time course assay in which log phase *sur7Δ* cells were incubated in 10 μM CuSO_4_ for the indicated time at 37°C and then stained with SYTOX Green. The *sur7Δ* cells suffered greater membrane damage as early as 30 min after addition of copper.(C) Log phase cells of the morphogenesis mutants *rvs161Δ*, *rvs167Δ*, and *arp2Δ arp3Δ* strains were incubated in 10 μM CuSO_4_ for 2 hr at 37°C and then stained with SYTOX Green. All mutants demonstrated increased staining, indicative of membrane permeabilization. (D) Log phase cells of known copper mutants were incubated in 10 μM CuSO_4_ for 2 hr at 37°C and then stained with SYTOX Green. The *crp1Δ*, *crp1Δ cup1Δ*, and *cup2Δ* strains all showed significantly less staining than the *sur7Δ* strain, indicating a low level of membrane permeabilization caused by CuSO_4_. Error bars indicate SD. ****, P<0.001. ***, P<0.01. **, P<0.01, *, P<0.05 by two-way ANOVA in comparison to the corresponding wild type values. The results represent the average of three independent assays (panels A, C, D) or six independent assays (Panel B) carried out on different days. Strains used included DIC185, *sur7Δ* (YJA11), *crp1Δ* (KC16), *cup1Δ crp1Δ* (KC25), and *cup2Δ* (YLD117-1).

The observation that *sur7Δ* cells have altered plasma membrane architecture, including mislocalization of cortical actin, septins, and the lipid PI_4,5_P_2_ [26, 31, 33], suggested the possibility that copper reduces the viability of these cells by permeabilizing the plasma membrane. Copper has been shown to permeabilize the plasma membranes of a wide range of cell types [38–41], but the mechanism is not known and no mutants have been described previously that are hypersensitive to this process. To examine how copper affects the plasma membrane barrier function, we incubated *C*. *albicans* cells with different concentrations of CuSO_4_ for 2 hr, and then treated the cells with the membrane impermeable dye SYTOX Green that can only stain intracellular nucleic acids if the plasma membrane has been compromised. Interestingly, the *sur7Δ* cells showed significantly increased staining in a dose-dependent manner compared to the wild type and *sur7Δ* + *SUR7* complemented strains (Fig 5A and S2 Fig). A time course analysis demonstrated that *sur7Δ* cells experienced more membrane damage than other strains even after a short 30 min incubation in copper (Fig 5B). Similar results were obtained with other membrane-impermeable stains, such as propidium iodide and FM4-64, and copper reduced the fluorescence of *sur7Δ* cells carrying the plasma membrane localized Pma1-GFP fusion protein, further supporting the conclusion that copper was permeabilizing the plasma membrane and having toxic effects (S3 Fig).

The other copper-sensitive morphogenesis mutants *rvs161Δ*, *rvs167Δ*, and *arp2Δ arp3Δ* showed strong SYTOX Green staining after incubation in copper, with *arp2Δ arp3Δ* exhibiting the highest level of staining (Fig 5C). This correlated with *arp2Δ arp3Δ* being the most copper-sensitive strain (Fig 4B). In contrast, the mutant strains that lack the Crp1 copper exporter or the Cup1 metallothionein that sequesters intracellular copper (*crp1Δ*, *cup1Δ crp1Δ*, and *cup2Δ*) showed only a small increase in SYTOX Green staining after copper treatment, which was significantly lower than that seen for *sur7Δ* and the other morphogenesis mutants (Fig 5D). This indicates that plasma membrane permeabilization by copper is separate from the toxic effects of intracellular copper.

**Fig 6:**
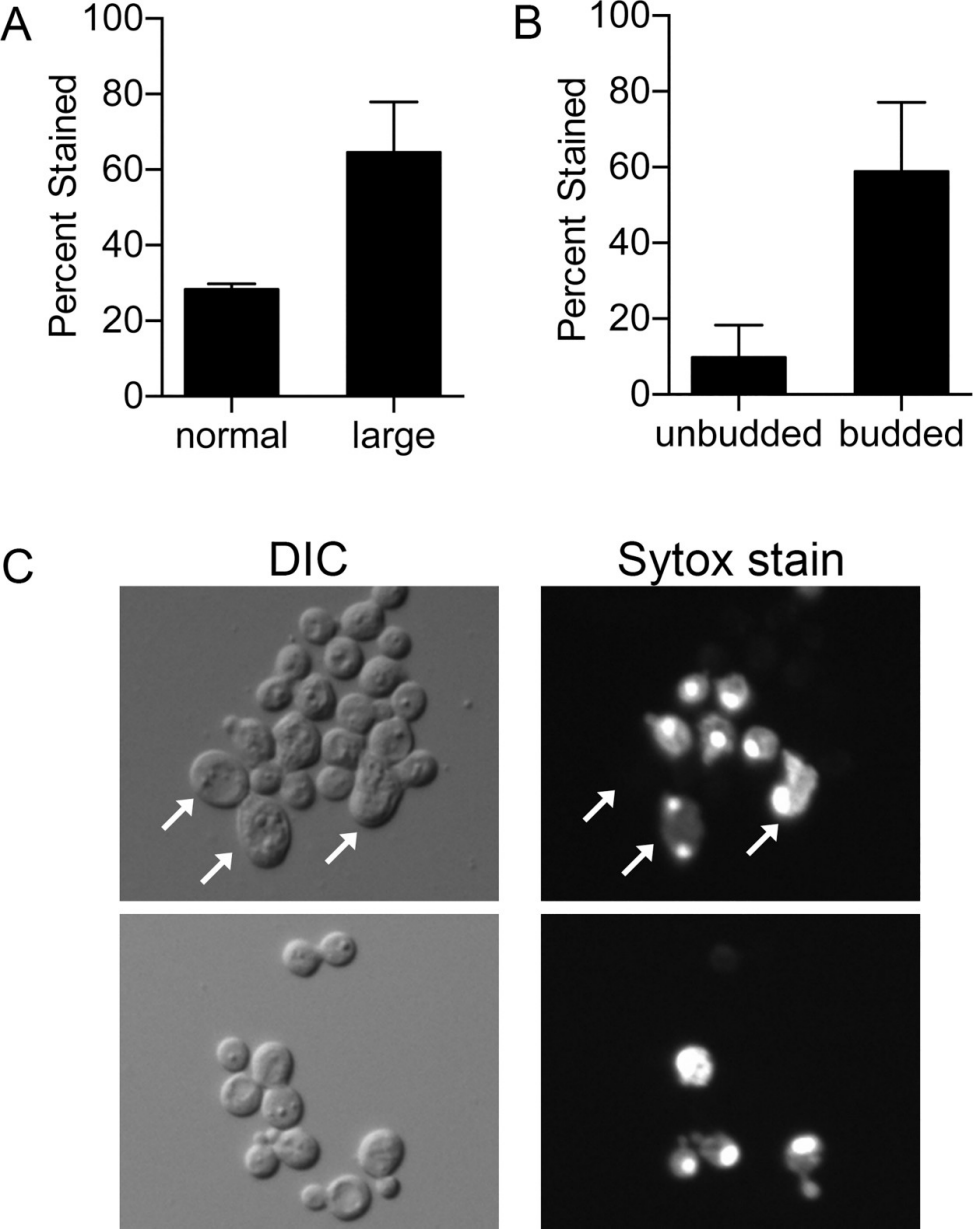
Cell morphology affects plasma membrane permeabilization by copper. Log phase cells were incubated in a solution of 10 μM CuSO_4_ for 2 hr at 37°C and then stained with SYTOX Green. (A) A greater percentage of large *sur7Δ* cells (≥ 7 μm) stained compared to those of normal size. (B) More budded *sur7Δ* cells stained with SYTOX Green than unbudded cells.(C) Representative photographs of SYTOX Green stained *sur7Δ* cells with arrows indicating large cells (>7 μm; upper right panel). Some cells of normal size also stained. Budded cells that stained are shown in the lower right panel. DIC images appear on left. The graphs represent averages of four independent experiments performed on different days. Strains included wild type DIC185 and *sur7Δ* (YJA11).

The relationship between cell morphology and SYTOX Green staining was examined since *sur7Δ* cells are known to range from typical-looking budding cells to abnormally large cells [33]. Interestingly, about 65% of large cells (≥ 7 μm) stained with SYTOX Green after copper treatment compared to only about 25% of normal sized cells (Fig 6A and 6C). Although the larger cells were permeabilized more readily, it is significant that the typical-sized cells were also susceptible to copper. The cell cycle stage of the *sur7Δ* cells also affected permeabilization by copper, as about 60% of budded cells stained compared to only 10% of unbudded cells (Fig 6B and 6C). This correlates with increased function of the actin cytoskeleton and vesicle trafficking during polarized bud morphogenesis.

### Increased susceptibility to antibiotics indicates copper-sensitive morphogenesis mutants have altered surface distribution of plasma membrane lipids

**Fig 7:**
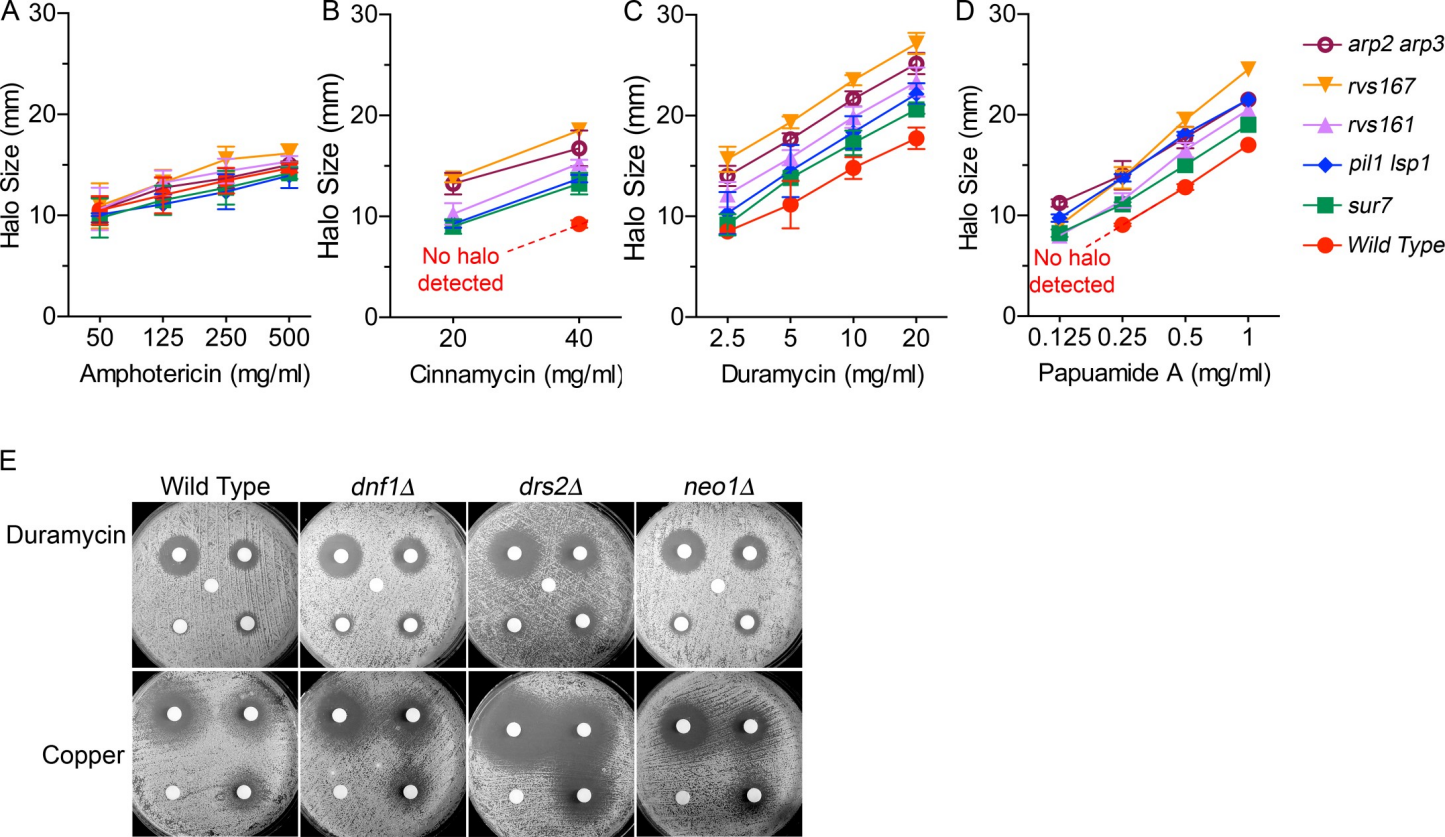
Altered responses of copper sensitive morphogenesis mutants and flippase mutants to membrane-targeted drugs. The susceptibility of copper-sensitive mutants to (A) amphotericin B, (B) cinnamycin, (C) duramycin and (D) papuamide A was determined by halo assays in which 2.5 x 10^5^ cells of the indicated strain were spread onto the surface of minimal medium plates, filter discs containing different amounts of the drugs were placed on the surface of each plate, and then the zone of growth inhibition was quantified. (E) Susceptibility of *C*. *albicans* flippase mutants to duramycin and copper. The filter disks on one plate contained 10 μl of 0, 2.5, 5, 10, or 20 mg/ml duramycin. The other plate contained 0, 75, 150, or 300 mM CuSO_4_. The zone of growth inhibition (halo) surrounding each filter disc was recorded after incubation for 48 hr at 30°C. Note that there were no significant differences in halo sizes between the copper-sensitive mutants and the wild type for amphotericin B which targets ergosterol, but significant differences were detected for cinnamycin and duramycin that target phosphatidylethanolamine (PE) and for papuamide A that targets phosphatidylserine (PS). Graphs represent averages of three independent experiments performed on different days. A representative set of halo assays is shown in the supporting information (S5 Fig). None of the mutants were significantly different from the wild type for amphotericin B, but all of the mutants were significantly different from the wild type for the other drugs by two-way ANOVA (P < 0.05 or lower). Strains in panels A-D were wild type control DIC185, *sur7Δ* (YJA11), *pil1Δ lsp1Δ* (YHXW21-1), *rvs161Δ* (YLD14-3), *rvs167Δ* (YLD16), and *arp2Δ arp3Δ* (CaEE27). Strains in Panel E were wild type control DIC185, and the flippase mutants included *neo1*Δ (C1_04630CΔ; YLD224-9), *dnf1*Δ (C5_00570WΔ, YLD219-2-9-1) and *drs2*Δ (C3_07230WΔ; YLD220-14-18-1).

The increased ability of copper to permeabilize the mutants suggested that their cell surfaces may be altered, so plasma membrane organization was probed by assaying sensitivity to compounds that target specific plasma membrane lipids. The *sur7Δ*, *pil1Δ lsp1Δ*, *rvs161Δ*, *rvs167Δ*, and *arp2Δ arp3Δ* mutants all showed essentially the same sensitivity as wild type cells to the polyene antibiotic amphotericin B (Fig 7A), which binds ergosterol in the plasma membrane [54]. In contrast, the mutant cells were more sensitive to the lanthionine antibiotics cinnamycin and duramycin (Fig 7B and 7C), which bind phosphatidylethanolamine (PE) if it is in the outer leaflet of the plasma membrane [55]. Similarly, all of the mutants were more susceptible to the depsipeptide antibiotic papuamide A (Fig 7D), which binds phosphatidylserine (PS) in the outer leaflet [56, 57]. PE and PS are normally enriched in the inner leaflet of the plasma membrane by proper membrane trafficking and the action of flippase proteins that promote inward translocation of these lipids [58]. The increased sensitivity to these drugs therefore indicates an altered surface of the plasma membrane. Interestingly, the relative increased susceptibility of the *sur7Δ*, *pil1Δ lsp1Δ*, *rvs161Δ*, *rvs167Δ*, and *arp2Δ arp3Δ* mutants to cinnamycin, duramycin, and papuamide A correlated with their relative sensitivity to permeabilization by copper, consistent with an altered presentation of lipids on the cell surface contributing to the ability of copper to permeabilize a membrane.

To confirm these results, we mutated members of the phospholipid flippase gene family in *C*. *albicans*, which encode P4-ATPases that promote inward translocation of lipids. Flippases are responsible for maintaining phospholipid asymmetry in the plasma membrane by translocating PS and PE from the outer leaflet to the inner leaflet of the plasma membrane [58]. Little or no analysis of flippases has been carried out in *C*. *albicans*, but in *S*. *cerevisiae NEO1* is essential and the other four have redundant functions (*DNF1*, *DNF2*, *DNF3* and *DRS2*). Surprisingly, although the apparent *C*. *albicans* ortholog of *NEO1* (C1_04630C) is not essential, a *neo1Δ* mutant showed a detectable increase in sensitivity to both duramycin and copper (Fig 7E). Similar results were obtained for the *dnf1Δ* (lacking C5_00570W). Interestingly, the *drs2Δ* mutant (lacking C3_07230W) showed the strongest increase in sensitivity to both duramycin, and copper. These results provide additional evidence that altered plasma membrane organization contributes to the increased sensitivity to copper.

To test whether the *sur7Δ* strain was altered in response to other agents that compromise plasma membrane integrity, we treated cells with the polycations DEAE dextran hydrochloride (500 kDa), and poly-L-lysine hydrobromide (30 kD). Poly-L-lysine with molecular weight > 25 kDa was reported to permeabilize yeast cells, as were DEAE dextrans with masses from 5 to 380 kDa [59]. The *sur7Δ* mutant showed slightly increased viability after exposure to either 0.1 μg/ml poly-L-lysine or 0.5 μg/ml DEAE dextran compared to the wild type control, indicating it was more resistant to this type of membrane disruption (S4 Fig) These results further confirm that copper has a specific effect on the *sur7Δ* plasma membrane.

Previous studies indicated that introducing polyunsaturated fatty acids (PUFAs) into the *S*. *cerevisiae* plasma membrane, which normally lacks PUFAs, increased the susceptibility to permeabilization by copper [60]. However, this did not seem likely to be a factor for *C*. *albicans*, as fatty acid analysis indicated that there were no significant differences in PUFA content in *C*. *albicans* strains between the wild type control, *sur7Δ*, and the *pil1Δ lsp1Δ* mutant (S1 Table). Furthermore, in *S*. *cerevisiae* the susceptibility to copper permeabilization plateaued at 20% PUFAs, and *C*. *albicans* naturally contains about 30% PUFAs [61].

### Phosphatidylserine (PS) promotes copper permeabilization of *sur7Δ* plasma membrane

**Fig 8:**
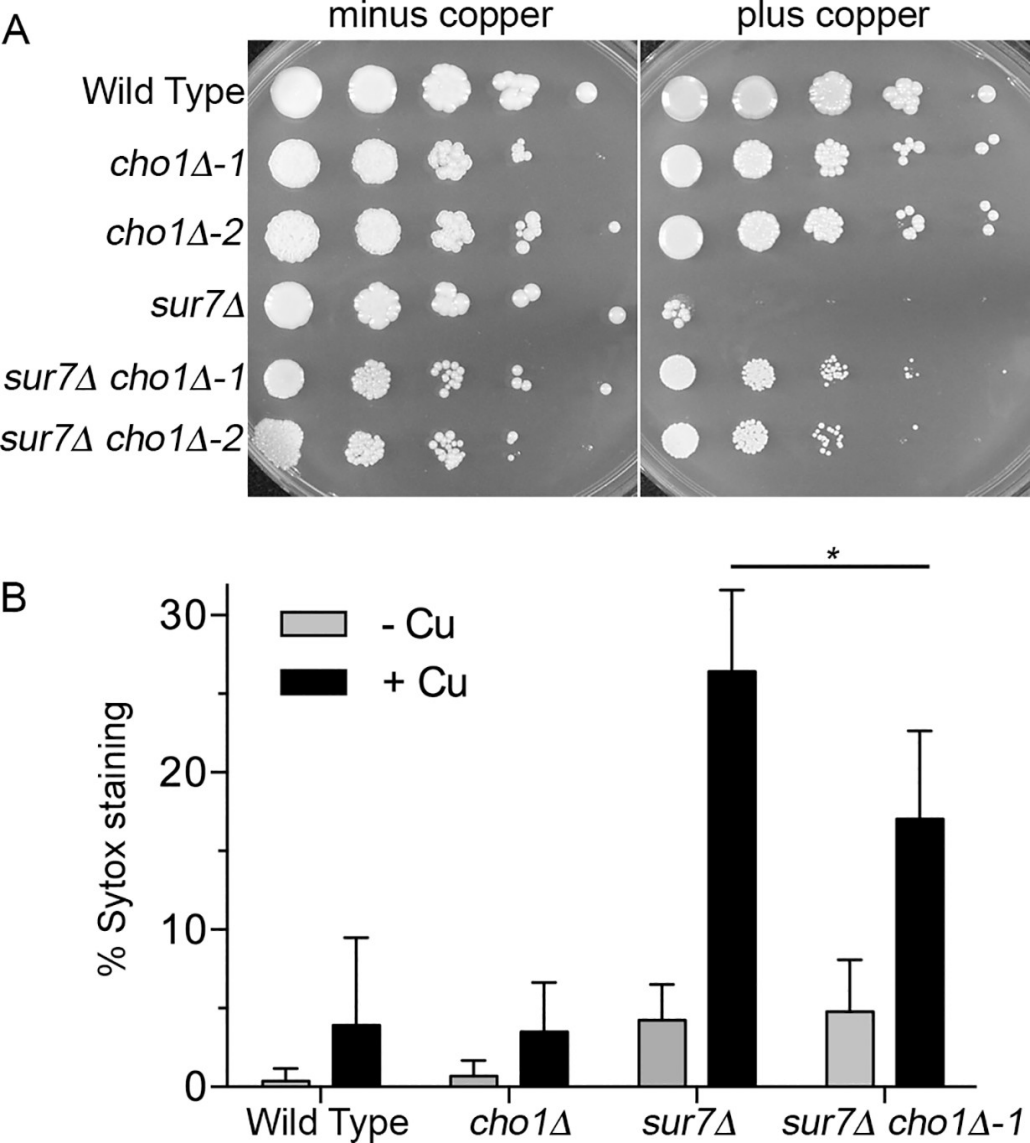
Deletion of the phosphatidylserine synthase gene *CHO1* rescues copper sensitivity of the *sur7Δ* mutant. (A) Dilutions of cells were spotted onto minimal synthetic medium in the absence or presence of 3 mM CuSO_4_ and incubated for 48 hr at 30°C. (B) The indicated cell types were incubated in water (- Cu) or 10 μM CuSO_4_ (+ Cu) for 2 hr at 37°C and then stained with SYTOX Green. The bars represent the average of at least 4 independent assays and error bars indicate SD. * indicates P < 0.02 by t test comparison. Strains used were wild type control DIC185, *cho1Δ* (YLD222-74 and YLD222-75), *sur7Δ* (YJA11), and *sur7Δ cho1Δ* (YLD221-5 and YLD221-6).

The increased sensitivity to papuamide indicated that PS was inappropriately exposed on the plasma membrane surface. Previous studies demonstrated that PS binds copper with very high affinity, and that this interaction can promote membrane damage that could lead to permeability [43, 44, 62]. To test the role of PS, we deleted the *CHO1* gene that encodes PS synthase [63]. However, the *sur7Δ cho1Δ* cells that lack PS were more resistant to copper than were the parental *sur7Δ* cells (Fig 8A). The *sur7Δ cho1Δ* double mutant consistently showed decreased permeabilization by copper relative to the *sur7Δ* single mutant (Fig 8B). Although there was some day to day variability in the total number of cells stained with Sytox Green, the percent of stained *sur7Δ cho1Δ* cells subtracted from the *sur7Δ* single mutant value averaged 9.9 + 2.3%. Altogether, these results indicate that abnormal plasma membrane organization leads to exposure of PS on the plasma membrane where it can be bound and attacked by copper.

## Discussion

### Sur7 carries out a novel role in copper resistance

Copper resistance is important for a wide range of bacterial and fungal pathogens [8, 11, 22, 64, 65]. Previous studies indicated that the increased sensitivity of *sur7Δ* mutant cells to copper-mediated killing was not due to a defect in the Crp1 copper exporter, which carries out the major known copper resistance mechanism in *C*. *albicans* [18, 19, 26]. Therefore, this study was initiated to better understand the role of the MCC/eisosome protein Sur7 in promoting resistance to copper. Analysis of a set of mutants lacking different MCC/eisosome proteins revealed that a *pil1Δ lsp1Δ* mutant, which is defective in forming the furrow-like membrane invaginations corresponding to MCC/eisosomes, was also more sensitive to copper (Fig 1). Sur7 is not stably maintained in the plasma membrane of *pil1Δ lsp1Δ* mutants [31], so we overexpressed *SUR7* and found that this strongly rescued the copper defect of the *pil1Δ lsp1Δ* cells, indicating that Sur7 plays the key role in copper resistance, rather than eisosome furrows that can form in the absence of Sur7. Further analysis of the *sur7Δ* cells showed that they were not highly sensitive to other metals (Fig 2), which indicates that the defect is specific to copper and not due to alteration in vacuolar function that is associated with increased sensitivity to multiple metals [20, 66, 67]. The effect of copper on the *sur7Δ* cells was not due to a general ability of copper to catalyze creation of reactive oxygen species, since the *sur7Δ* mutant did not show a correspondingly large increase in susceptibility to oxidative stress (Fig 3). These results indicate that Sur7 carries out a novel role in copper resistance.

### Genetic approaches link copper sensitivity to altered plasma membrane organization and phosphatidylserine

Genetic screening of *C*. *albicans* mutants was used to gain further insight into the mechanisms underlying the increased copper sensitivity of *sur7Δ* cells. Interestingly, most of the copper-sensitive mutants that were identified have defects in membrane organization or trafficking (Fig 2). Targeted analysis of three mutants known to have strong defects in plasma membrane function and cortical actin (*rvs161Δ*, *rvs167Δ*, and *arp2Δ arp3Δ* mutants) revealed that they all showed increased susceptibility to killing by copper that was similar to or greater than the *sur7Δ* mutant (Fig 4). In support of a role for the actin cytoskeleton, wild type cells treated with the actin inhibitor cytochalasin A also displayed increased sensitivity to copper (Fig 4C). Furthermore, overexpression of *SUR7*, which rescued the copper defect of *pil1Δ lsp1Δ* cells (Fig 1B), was shown in a previous study to improve plasma membrane organization of cortical actin [31]. Altogether, these results indicate that altered plasma membrane organization caused by defects in the MCC/eisosome protein Sur7 or disruption of the actin cytoskeleton increases the susceptibility to killing by copper.

Copper binds strongly to membranes, which suggested it could have a direct effect on lipids. In particular, copper binds to PS with picomolar affinity and to PE with micromolar affinity [43, 44]. Copper is thought to bind PS with such high affinity relative to other divalent metals because it can interact in a special configuration with two molecules of PS [42–44]. Usually PS and PE are sequestered on the inner leaflet of the plasma membrane by the action of phospholipid flippases [68]. To determine whether these phospholipids were exposed on the cell surface of the copper-sensitive mutants, the mutant cells were tested for increased sensitivity to cinnamycin and duramycin, which bind to PE, and to papuamide A, which binds to PS (Fig 7). There was a good correlation between the relative increases in sensitivity to copper and to these drugs that target PS and PE, consistent with copper sensitivity reflecting the degree to which the surface exposure of PS and PE is altered in the mutants. The mutants that were most susceptible to being permeabilized by copper (*sur7Δ*, *pil1Δ lsp1Δ*, *rvs161Δ*, *rvs167Δ*, and *arp2Δ arp3Δ*) all had broad defects in plasma membrane structure that could result in the cell surface exposure of PS and PE [33, 52, 53, 69]. Furthermore, three different phospholipid flippase deletion mutants displayed increased sensitivity to copper, especially the *drs2Δ* mutant (Fig 7). We therefore examined the effects of deleting the gene encoding PS synthase (*CHO1*) to deplete cells of PS. Remarkably, the *sur7Δ cho1Δ* double mutant showed greatly improved resistance to copper (Fig 8). These results strongly support the conclusion that copper has a direct effect on the plasma membrane, and implicate interaction with PS as part of the underlying mechanism for copper-mediated killing of the mutant cells.

### Mechanisms of plasma membrane permeabilization and killing by copper

Although plasma membrane permeabilization can be a consequence of cell death, several lines of evidence indicate that copper kills *sur7Δ* cells and the other new copper-sensitive mutants we identified by promoting membrane permeabilization. Previous studies have shown that copper is distinct from other divalent metal ions in its ability to bind and damage model membranes containing PS or PE [42–44]. Atomic force microscopy studies showed copper promotes lipid reorganization and affects membrane fluidity that could weaken the membrane barrier function [70, 71]. The ability of excess copper to permeabilize red blood cell membranes was correlated with a loss of membrane fluidity and decreased ability of the cells to tolerate deformation [72]. Copper has been known to permeabilize the plasma membranes of fungi and other cell types from plants to animals for 30 years, and has been used as a tool to form pores in the plasma membrane to selectively release cytoplasmic pools of amino acids [36, 38–41], but the mechanism by which copper permeabilizes the plasma membrane is not known. It is therefore significant that the *sur7Δ*, *pil1Δ lsp1Δ*, *rvs161Δ*, *rvs167Δ*, and *arp2Δ arp3Δ* mutants represent the first mutants reported for being more susceptible to copper permeabilization (Fig 5), as they provide new insights into the mechanisms underlying this process. Once cells become permeabilized, copper would be expected to enter cells and cause further lethal damage. In contrast, control studies showed that copper did not cause a significant increase in plasma membrane permeability for the *crp1Δ*, *cup1Δ crp1Δ*, and *cup2Δ* mutants in spite of their greatly increased sensitivity to this metal (Fig 5D). This supports the conclusion that there are independent pathways for killing by copper.

The special redox properties of copper suggest that it damages membranes by promoting oxidation. Studies with copper-treated model membranes demonstrate that this metal can promote oxidation of both PS and PE [42, 44]. In our studies, the oxidation-sensitive *C*. *albicans* mutant *pst1Δ pst2Δ pst3Δ ycp4Δ* mutant (Fig 1A) and a *cat1Δ* catalase mutant (Fig 3) did not show a significant increase in susceptibility to copper. However, it may be that these intracellular proteins do not have a direct effect on oxidation events in the outer leaflet of the plasma membrane. Furthermore, other data showed that the oxidative effects of copper on PS are differentially affected by different subsets of anti-oxidant molecules, indicating that not all antioxidant pathways are likely to be equally effective [62]. Thus, copper may be acting in a specialized microenvironment at the cell surface to promote oxidative damage to membranes leading to permeabilization.

### Potential roles of copper in antifungal therapy

The plasma membrane is an effective target for therapy, as the commonly used antifungal drugs alter the plasma membrane directly or indirectly (i.e. fluconazole, amphotericin, caspofungin) and many antimicrobial peptides act by permeabilizing the plasma membrane [54, 73]. One reason the antifungal drugs are effective is that even at sublethal doses they can broadly impact plasma membrane organization [74]. Proper architecture of proteins and lipids is required to form a strong protective barrier around the cell, and it plays key roles in virulence by coordinating a wide range of dynamic functions including secretion of virulence factors, cell wall synthesis, invasive hyphal morphogenesis, endocytosis, and nutrient uptake [74]. The identification in this study of new copper-sensitive mutants and the role of phosphatidylserine provides insights for new modes of antifungal therapy. Copper is amenable to incorporation into therapeutic strategies [75, 76]. Drugs that alter the plasma membrane surface may be used to increase the susceptibility of cells to copper in the host [6–10], and conversely copper could be used to enhance the membrane perturbing effects of currently used antifungal drugs [77]. In addition, our results have important implications for understanding microbial killing by metallic copper surfaces, which have been explored for preventing the spread of nosocomial infections [45, 46]. Thus, copper could be used in a synergistic fashion with other therapeutic strategies to more effectively kill fungal pathogens.

## Materials and methods

### Strains and media

The *C*. *albicans* strains used in this study appear in Table 1. Cultures were grown either in rich YPD medium (2% dextrose, 1% peptone, 2% yeast extract, 80 mg/L uridine), or synthetic medium (yeast nitrogen base, 2% dextrose) [78]. Synthetic medium was supplemented with amino acids and uridine, if required. The *pbs2Δ*, *ssk2Δ*, *hog1Δ*, *cup2Δ*, and *neo1Δ* strains [47] were made prototrophic by transformation with a PCR-amplified *ARG4* DNA fragment that was integrated into the native locus. Primers used to amplify the cassette included 18 base pairs of homology approximately 500 base pairs upstream and downstream of the *ARG4* open reading frame. The *sla2Δ*, *vps28Δ*, *vps16Δ*, *cka2Δ*, *ckb2Δ*, *and ssn8Δ* strains [48] were transformed with a similarly constructed *HIS1* cassette to correct the remaining auxotrophy. PMA1-GFP strains were constructed with the photostable GFPγ variant, as described previously [79]. Homozygous *dnf1*Δ (orf19.932/C5_00570W) and *drs2*Δ (19.6778/ C3_07230W) mutants were constructed from *C*. *albicans* parental strain BWP17 by the sequential deletion of both copies of the targeted gene [80]. Deletion cassettes were generated by PCR amplification of the *ARG4* or *HIS1* selectable marker genes, using primers that included 70 to 80 base pairs of DNA sequence homologous to the upstream or downstream regions of the targeted open reading frame. Cells which had undergone homologous recombination to delete the targeted gene were identified by PCR analysis using a combination of primers flanking sites of cassette integration and internal primers. Homozygous *cho1*Δ mutants were constructed in *C*. *albicans* parental strain DIC185 and the previously described *sur7*Δ deletion (33), using the transient CRISPR-Cas9 system [81]. The gene-deletion construct was synthesized using the *SAT1* flipper as a template and primers that included 80 base pairs upstream and downstream of the targeted gene [82]. Homozygous deletion clones were identified by rescue of the ethanolamine auxotrophy phenotype on synthetic medium with 1mM ethanolamine [63], and by PCR analysis using primers flanking the *CHO1* gene, as well as *SAT1* internal primers.

**Table 1:** *C*. *albicans* strains used in this study.

Strain	Short genotype	Full genotype
BWP17	parental strain	*his1*::*hisG/his1*::*hisG arg4*::*hisG/arg4*::*hisG ura3*::*λimm434/ ura3*::λ*imm434*
DIC185	prototrophic WT control	*ura3*::*λimm434/URA3 his1*::*hisG/ HIS1 arg4*::*hisG/ ARG4*
YJA11	*sur7Δ*	*sur7Δ*::*ARG4/sur7Δ*::*HIS1 URA3/ura3*::*λimm434 his1*::*hisG/his1*::*hisG arg4*::*hisG/arg4*::*hisG*
YJA12	*sur7Δ SUR7*	*sur7Δ*::*ARG4/sur7Δ*::*HIS1 SUR7*::*URA3 ura3*::*λimm434/ ura3*::*λimm434 his1*::*hisG/his1*::*hisG arg4*::*hisG/arg4*::*hisG*
YHXW21-1	*pil1Δ lsp1Δ*	*pil1Δ*::*ARG4/ pil1Δ*::*FRT lsp1Δ*::*HIS1/lsp1Δ*::*SAT1 flipper URA3/ura3*::*λimm434 his1*::*hisG/his1*::*hisG arg4*::*hisG/arg4*::*hisG*
YHXW23-1	*pil1Δ lsp1Δ LSP1*	*pil1Δ*::*ARG4/pil1Δ*::*FRT lsp1Δ*::*HIS1/lsp1Δ*::*SAT1 flipper ura3*::*λimm434/ ura3*::*λimm434 his1*::*hisG/his1*::*hisG arg4*::*hisG/arg4*::*hisG LSP1*::*URA3*
YLD182-9-10-2	*seg1Δ*	*seg1Δ*::*ARG4/seg1Δ*::*HIS1 URA3/ura3*::*λimm434 his1*::*hisG/his1*::*hisG arg4*::*hisG/arg4*::*hisG*
YHXW36-1	*slm2Δ*	*orf19*.*3505Δ*::*ARG4/ orf19*.*3505Δ*::*HIS1 URA3/ ura3*::*λimm434 his1*::*hisG/his1*::*hisG arg4*::*hisG/arg4*::*hisG*
YLD146-11-2-5	*pkh1Δ pkh3Δ PKH3*	*pkh1Δ*::*ARG4/ pkh1Δ*::*HIS1 pkh3Δ*::*FRT/ PKH3 URA3/ura3*::*λimm434 his1*::*hisG/his1*::*hisG arg4*::*hisG/arg4*::*hisG*
LLF60A	*pst1Δ pst2Δ pst3Δ ycp4Δ*	*pst3-ycp4Δ*::*LEU2/pst3-ycp4Δ*::*HIS1pst2Δ*::*FRT/pst2Δ*::*FRT pst1Δ*::*FRT/pst1Δ*::*FRT ARG4/arg4Δ*
YHXW14	*nce102Δ*	*nce102Δ*::*ARG4/ nce102Δ*::*HIS1 URA3/ ura3*::*λimm434 his1*::*hisG/his1*::*hisG arg4*::*hisG/arg4*::*hisG*
YLD204-2	*pun1Δ*	*pun1Δ*::*HIS1/pun1Δ*::*LEU2 his1Δ/his1Δ leu2Δ/leu2Δ ARG4/arg4Δ URA3/ura3*::*imm IRO1/iro1Δ*::*imm*
YHXW3	*fmp45Δ*	*fmp45Δ*::*ARG4/fmp45Δ*::*HIS1 URA3/ ura3*::*λimm434 his1*::*hisG/his1*::*hisG arg4*::*hisG/arg4*::*hisG*
KC25	*cup1Δ crp1Δ*	*cup1Δ*::*hisG/cup1Δ*::*hisG crp1Δ*::*hisG/crp1Δ*::*hisG-URA3-hisG ura3*::*imm434/ura3*::*imm434*
YHXW45-1	*pil1Δ lsp1Δ 1xSUR7*	*pil1Δ*::*ARG4/pil1Δ*::*FRT lsp1Δ*::*HIS1/lsp1Δ*::*FRT ura3*::*λimm434/ura3*::*λimm434 his1*::*hisG/his1*::*hisG arg4*::*hisG/arg4*::*hisG SUR7*::*URA3*
YHX46-1	*pil1Δ lsp1Δ 2xSUR7*	*pil1Δ*::*ARG4/pil1Δ*::*FRT lsp1Δ*::*HIS1/lsp1Δ*::*FRT ura3*::*λimm434/ura3*::*λimm434 his1*::*hisG/his1*::*hisG arg4*::*hisG/arg4*::*hisG SUR7*::*URA3 SUR7*::*NAT1*
YLD197-1	*pbs2*	*pbs2Δ*::*HIS1/pbs2Δ*::*LEU2 his1Δ/his1Δ leu2Δ/leu2Δ ARG4/arg4Δ URA3/ura3*::*imm IRO1/iro1Δ*::*imm*
YLD185-7	*ssk2Δ*	*ssk2Δ*::*HIS1/ssk2Δ*::*LEU2 his1Δ/his1Δ leu2Δ/leu2Δ ARG4/arg4Δ URA3/ura3*::*imm IRO1/iro1Δ*::*imm*
YLD184-3	*hog1Δ*	*hog1Δ*::*HIS1/hog1Δ*::*LEU2 his1Δ/his1Δ leu2Δ/leu2Δ ARG4/arg4Δ URA3/ura3*::*imm IRO1/iro1Δ*::*imm*
YLD14-3	*rvs161Δ*	*rvs161Δ*::*ARG4/rvs161Δ*::*HIS1 URA3/ura3*::*λimm434 his1*::*hisG/his1*::*hisG arg4*::*hisG/arg4*::*hisG*
YLD16-11	*rvs167Δ*	*rvs167Δ*::*ARG4/rvs167Δ*::*HIS1 URA3/ura3*::*λimm434 his1*::*hisG/his1*::*hisG arg4*::*hisG/arg4*::*hisG*
YLD193-10	*sla2Δ*	*sla2*::*URA3/sla2*::*URA3-ARG4-URA3 HIS1/his1*::*hisG arg4*::*hisG/arg4*::*hisG ura3*::*λimm434/ ura3*::*λimm434*
YLD187-11	*pep7*	*pep7Δ*::*HIS1/pep71Δ*::*LEU2 his1Δ/his1Δ leu2Δ/leu2Δ ARG4/arg4Δ URA3/ura3Δ*::*imm IRO1/iro1Δ*::*imm*
YLD188-1	*och1*	*och1Δ*::*HIS1/och1Δ*::*LEU2 his1Δ/his1Δ leu2Δ/leu2Δ ARG4/arg4Δ URA3/ura3*::*imm IRO1/iro1Δ*::*imm*
YLD200-2	*vps28Δ*	*vps28*::*URA3/vps28*::*URA3-ARG4-URA3 HIS1/his1*::*hisG arg4*::*hisG/arg4*::*hisG ura3*::*λimm434/ura3*::*λimm434*
YLD186-1	*cne1*	*cne1Δ*::*HIS1/cne1Δ*::*LEU2 his1Δ/his1Δ leu2Δ/leu2Δ ARG4/arg4Δ URA3/ura3*::*imm IRO1/iro1Δ*::*imm*
YLD191-4	*stt4*	*stt4Δ*::*HIS1/stt4Δ*::*LEU2 his1Δ/his1Δ leu2Δ/leu2Δ ARG4/arg4Δ URA3/ura3*::*imm IRO1/iro1Δ*::*imm*
YLD202-1	*vps16Δ*	*vps16*::*URA3/vps16*::*URA3-ARG4-URA3 HIS1/his1*::*hisG arg4*::*hisG/arg4*::*hisG ura3*::*λimm434/ura3*::*λimm434*
YLD194-2	*cka2*	*cka2*::*URA3/cka2*::*URA3-ARG4-URA3 HIS1/his1*::*hisG arg4*::*hisG/arg4*::*hisG ura3*::*λimm434/ura3*::*λimm434*
YLD195-1	*ckb2*	*ckb2Δ*::*URA3/ckb2*::*URA3-ARG4-URA3 HIS1/his1*::*hisG arg4*::*hisG/arg4*::*hisG ura3*::*λimm434/ura3*::*λimm434*
YLD196-7	*ssn8*	*ssn8*::*URA3/ssn8*::*URA3-ARG4-URA3 HIS1/his1*::*hisG arg4*::*hisG/arg4*::*hisG ura3*::*λimm434/ ura3*::*λimm434*
YLD115-1	*crp1*	*URA3*::*λimm434/ura3Δ*::*λimm434 crp1*::*hisG/crp1*::*hisG*
YLD117-1	*cup2*	*cup2Δ*::*HIS1/cup2Δ*::*LEU2 his1Δ/his1Δ leu2Δ/leu2Δ ARG4/arg4Δ URA3/ura3Δ*::*imm IRO1/iro1Δ*::*imm*
YLD116-7	*cup1Δ*	*URA3/ura3Δ*::*λimm434 cup1Δ*::*hisG/cup1Δ*::*hisG*
MT505-A	*cat1Δ*	*cat1Δ*::FRT/*cat1Δ*::FRT
CaEE227	*arp2Δ arp3Δ*	*arp2*::*LEU2/arp2*::*HIS1 arp3*::*URA3/arp3*::*ARG4*
KC16	*crp1Δ*	*ura3*::*imm434* /*ura3*::*imm434 crp1Δ*::*hisG/crp1Δ*::*hisG*
YHXW11	*PMA1-GFPγ*	*ura3*::*λimm434/ ura3*::*λimm434 PMA1-GFPγ*::*URA3*
YHXW61	*sur7Δ PMA1-GFPγ*	*sur7Δ*::*ARG4/ sur7Δ*::*HIS1 ura3*::*λimm434 /ura3*::*λimm434 his1*::*hisG/his1*::*hisG arg4*::*hisG/arg4*::*hisG PMA1-GFPγ*::*URA3*
YLD219-2-9-1	*dnf1Δ*	*dnf1Δ*::*ARG4/dnf1Δ*::*HIS1 URA3/ura3*::*λimm434 his1*::*hisG/his1*::*hisG arg4*::*hisG/arg4*::*hisG*
YLD220-14-18-1	*drs2Δ*	*drs2Δ*::*ARG4/drs2Δ*::*HIS1 URA3/ura3*::*λimm434 his1*::*hisG/his1*::*hisG arg4*::*hisG/arg4*::*hisG*
YLD224-9	*neo1Δ*	*neo1Δ*::*HIS1/dnf1Δ*::*LEU2 his1Δ/his1Δ leu2Δ/leu2Δ ARG4/arg4 URA3/ura3*:: *imm IRO1/iro1Δ*::*imm*
YLD221-5	*sur7Δ cho1Δ*	*sur7Δ*::*ARG4/sur7Δ*::*HIS1 URA3/ura3*::*λimm434 his1*::*hisG/his1*::*hisG arg4*::*hisG/arg4*::*hisG cho1Δ*::*FRT/cho1Δ*:: *SAT1 flipper*
YLD221-6	*sur7Δ cho1Δ*	*sur7Δ*::*ARG4/sur7Δ*::*HIS1 URA3/ura3*::*λimm434 his1*::*hisG/his1*::*hisG arg4*::*hisG/arg4*::*hisG cho1Δ*::*FRT/cho1Δ*:: *SAT1 flipper*
YLD222-74	*cho1Δ*	*ura3*::*λimm434/URA3 his1*::*hisG/ HIS1 arg4*::*hisG/ ARG4 cho1Δ*::*FRT/cho1Δ*:: *SAT1 flipper*
YLD222-75	*cho1Δ*	*ura3*::*λimm434/URA3 his1*::*hisG/ HIS1 arg4*::*hisG/ ARG4 cho1Δ*::*FRT/cho1Δ*:: *SAT1 flipper*

### Screening of mutant strains for sensitivity to copper and other metals

Mutant strain libraries of S. Noble [47], A. Mitchell [48], and O. Homann [23], along with strains in our own collection were replica-plated onto synthetic agar medium containing 500 μM, 250 μM, 100 μM, 20 μM, or 0 μM CuSO_4,_ plus added arginine, histidine, or uridine, if necessary. Other metals were tested similarly by replica-plating onto solid agar medium containing 1.5 M NaCl, 1.5 M KCl, 150 mM LiCl, 800 mM RbCl, 300 mM CsCl, 3 mM CoCl_2_, 300 μM CdCl_2_, 100 mM MnCl_2_, 20 mM ZnCl_2_, 4 mM NiCl_2_, 2 mM FeSO_4_, 200 μM CrO_3_, and 500 mM CaCl_2_. The concentrations of these metals were determined by identifying a dose at which the growth of wild type cells was reduced, but not lethal. Plates were incubated at 30°C for 48-72 hr, and then the extent of growth inhibition was recorded daily on a scale of 0 to 5, with 0 indicating the level of growth of the wild type strain and 5 indicating no growth. The results represent the average of at least two assays for screening each of the different metals, and then the susceptible mutants were confirmed in additional assays. The mutant strains were clustered using the program Cluster, and then a heat map was generated. The mutants that were not detectably sensitive to copper are listed in the Supplementary Information (S2 Table).

### Assays for viability and sensitivity to copper

Strains were grown overnight at 30°C in synthetic medium. Cells were then diluted to 5 x 10^3^ cells/ml in synthetic medium and 300 μl was added to the first row of wells of a 96-well plate (Costar, Corning, Inc., Kennebunk, ME). 240 μl of cells was applied to subsequent rows of the plate. CuSO_4_ was then added to wells of the first row to a final concentration of 10 mM and serial dilutions were made by transferring 60 μl from one row to the next. Plates were then covered with AeraSeal oxygen permeable sealing film and incubated at 37°C for 48 hr. The highest concentration of copper that supported growth was recorded. Similar methods were used to quantify the sensitivity of *C*. *albicans* strains to other metals. Viability following exposure to copper was assayed by growing strains overnight in synthetic medium to log phase at 30°C. The cells were then washed with sterile H_2_O and diluted to 3.0 x 10^3^ cells/ml in sterile 1 mM MES buffer pH 6 in the presence or absence of 0.5 μM CuSO_4_. Cells were then incubated at 37°C for the indicated time, 100 μl of each sample was then spread onto YPD agar plates, incubated at 30°C for 48 hr, and then CFUs were quantified. The effects of the actin polymerization inhibitor cytochalasin A (Sigma-Aldrich, St. Louis, MO) were examined with cells that were grown overnight in synthetic medium at 30°C to log phase, diluted to 1 x 10^6^ cells/ml in synthetic medium, and then cytochalasin A was added to a final concentration of 10 μg/ml every 30 min for 2 hr, with incubation at 30°C. Cytochalasin was added every 30 min to maintain a high level of this drug as it is not stable under these conditions [83]. Cells were then washed with sterile H_2_O, diluted to 3 x 10^3^ cells/ml in sterile H_2_O, a final aliquot of cytochalasin A was added, and cells were incubated with the indicated concentrations of CuSO_4_ for 2 hr at 37°C. 100 μl of each sample was then spread onto YPD agar plates and incubated at 30°C for 48 hr to determine the viable CFUs. Statistical analysis of the data was carried out using Prism software (GraphPad Inc.).

### Fluorescence microscopy

SYTOX Green (Invitrogen, Molecular Probes, Eugene, OR) is a membrane impermeable nucleic acid stain that can be used to assay plasma membrane integrity [84]. For the analysis, strains were grown in synthetic medium overnight at 30°C to log phase, washed in sterile H_2_O, and diluted to 1 x 10^7^ cells/ml. Following incubation in CuSO_4_ with 1 mM MES buffer pH 6 at 37°C, cells were washed in sterile H_2_O, SYTOX Green was added to a final concentration of 2.5 nM, and cells were incubated at room temperature for 5 min. Cells were washed again in sterile H_2_O and then analyzed by fluorescence microscopy. Propidium iodide (Invitrogen, Molecular Probes, Eugene, OR) staining was carried out in a similar manner, except the stain was added to a final concentration of 4.2 μg/ml, and incubation was carried out for 15 min at room temperature. Cells were stained with the lipophilic dye FM4-64 (Invitrogen, Molecular Probes, Eugene, OR) by adding it to a final concentration of 20 μM [85]. Cells were incubated on ice for 20 min, washed in cold sterile H_2_O, and viewed immediately by fluorescence microscopy. Images were obtained using an Olympus BH2 microscope (Melville, NY) equipped with a Zeiss AxioCam digital camera (Thornwood, NY).

### Antifungal drug growth inhibition assays

Cells were grown overnight in synthetic medium at 30°C, diluted to 1.0 x 10^6^ cells/ml in synthetic medium, and 250 μl was spread onto the surface of a synthetic medium agar plate. Paper filter disks (Becton Dickinson and Company, Sparks, MD) containing 10 μl of the concentration of drug to be assayed were applied onto the surface of plates containing the indicated strains. After incubation for 48 hr at 30°C, the diameters of the zones of growth inhibition were measured. Drugs tested included duramycin (Sigma Aldrich, St. Louis, MO), cinnamycin (Santa Cruz Biotechnology, Santa Cruz, CA), papuamide A (Flintbox, British Columbia, Canada), and amphotericin B (Sigma-Aldrich, St. Louis, MO). Determination of sensitivity to the oxidizing agent H_2_O_2_ (Fisher Scientific, Fair Lawn, NJ), was performed by spot assay. Cells grown overnight in synthetic medium at 30°C were diluted to 1.0 x 10^7^ cells/ml in synthetic medium and a 10-fold dilution series was made. 2 μl of each dilution was spotted onto YPD containing the indicated concentrations of H_2_O_2_ and plates were incubated at 30°C for 48 hr and then photographed. Strains tested for H_2_O_2_ sensitivity were also spotted onto SD medium containing CuSO_4_ for confirmation of copper sensitivity. To assay cells for reaction to polycations, strains were grown overnight in synthetic medium, washed in H_2_O, diluted to 3 x 10^3^ cells/ml in H_2_O, and then incubated with DEAE dextran hydrochloride (500 KDa, Sigma-Aldrich, St. Louis, MO) or poly-L-lysine hydrobromide (30 kDa, Sigma-Aldrich, St. Louis, MO) for 2 hr at 37°C. 100 μl of each sample was then spread onto YPD medium, incubated at 30°C for 48 hr, and then CFUs were quantified to assess viability.

### Fatty acid analysis

Cells were grown overnight in synthetic medium at 30°C, diluted to 1.0 x 10^6^ cells/ml in 50 ml synthetic medium, and then incubated with shaking at 37°C until a concentration of approximately 1.0 x 10^7^ cells/ml. Cells were then harvested by centrifugation, washed in sterile H_2_O, and then frozen cell pellets were shipped to Microbial ID, Inc. (Newark, DE) for fatty acid analysis. Sample preparation involved saponification, methylation, and extraction of fatty acid methyl esters. Gas chromatography was used to create a cellular fatty acid profile identifying the percent of each fatty acid species and localization of unsaturated bonds. For a second analysis, cells were streaked onto YPD medium, incubated at 30°C, and then submitted to Microbial ID, Inc. for testing.

## Supporting information

S1 Fig Loss of viability in the presence of copper at low pH. *C*. *albicans* strains were incubated in a solution of CuSO_4_ for the indicated time, and then the viability of the cells was determined by plating cells on YPD medium and counting the CFUs. This assay was similar to that shown in Fig 1D, except that HCl was used to lower the pH. The results represent the average of three independent experiments performed on different days. Error bars indicate SD. Strains used were: WT, wild-type DIC185; *sur7Δ* (YJA11); *sur7Δ* comp. (YJA12); *pil1Δ lsp1Δ* (YHXW21-1); *pil1Δ lsp1Δ* comp (+ *LSP1*, YHXW23-1); and *cup1Δ crp1Δ* (KC25).
(TIF)

S2 Fig SYTOX Green staining of copper-permeabilized cells. Representative samples of cells stained with the membrane impermeable dye SYTOX Green. Wild type and *sur7Δ* cells were incubated in the presence of 10 μM CuSO_4_ with 1 mM MES buffer at pH 6 for two hr. Cells were then washed, stained with SYTOX Green for 5 min, and then viewed by fluorescence microscopy. WT, wild-type DIC185; *sur7Δ* (YJA11).
(TIF)

S3 Fig Copper permeabilization of the plasma membrane results in loss of Pma1-GFP fluorescence and increased staining by propidium iodide and FM4-64. (A) Log phase *sur7Δ* cells engineered to produce a fusion between the plasma membrane H+ ATPase Pma1 and GFP were incubated in water or 10 μM CuSO_4_ for 2 hr at 37°C. Cells were then stained with the membrane-impermeable dye propidium iodide (PI). The graph depicts how copper treatment causes a loss in GFP fluorescence and an increase in membrane permeability, indicated by PI staining.
(B) Photographs showing that *sur7Δ PMA1-GFP* cells that lost the GFP signal with CuSO_4_ treatment stained with PI. The graph represents averages of three independent experiments performed on different days. Strains used were the wild type control *PMA1*- *GFPγ* (YHXW11) and *sur7Δ PMA1-GFPγ* (YHXW61).
(C) The *sur7Δ* strain *PMA1-GFP* (YHXW61) was incubated in the presence or absence of 10 μM CuSO_4_ with 1 mM MES buffer at pH 6 for two hr, stained with FM4-64, and then imaged by fluorescence microscopy. Note that loss of Pma1-GFP correlated with intense staining by FM4-64.
(TIF)

S4 Fig The *sur7Δ* strain does not exhibit increased susceptibility to killing by the membrane disrupting agents DEAE dextran or poly-lysine. The indicated strains were incubated with DEAE dextran hydrochloride (500 kDa) or poly-L-lysine hydrobromide (30 kDa) for 2 hr at 37°C. Samples were then plated onto YPD medium, incubated at 30°C for 48 hr, and then CFUs were counted to assess viability. WT, wild-type DIC185; *sur7Δ* (YJA11).
(TIF)

S5 Fig Samples of halo assays to determine the relative sensitivity of *C*. *albicans* strains to agents that target the plasma membrane. Representative halo assay for testing the sensitivity of cells to different drugs. A lawn of 2.5 x 10^5^ cells was spread on the surface of a synthetic medium agar plate, and then paper filter disks containing 10 μl of different concentrations of the drug were applied to the surface of the plate. After incubation for 48 hr at 30°C, the plates were photographed. Concentrations used for amphotericin were 500, 250, 125, 50, and 0 μg/ml. Concentrations used for cinnamycin were 40, 20, and 0 μg/ml. Concentrations used for duramycin were 20, 10, 5, and 2.5 μg/ml and 0 μg/ml. Concentrations used for papuamide A were 1000, 500, 250, 125, and 0 μg/ml. Strains used were DIC185, *sur7Δ* (YJA11), *rvs161Δ* (YLD14-3), *rvs167Δ* (YLD16), and *arp2Δ arp3Δ* (CaEE27)
(TIF)

S1 Table Fatty acid analysis. (DOCX)

S2 Table Mutant strains not detectably hypersensitive to copper. (XLSX)
