# Supplementary figures and images for: Plasma membrane architecture protects Candida albicans from killing by copper
Source: PLoS Genet. 2019 Jan 11;15(1):e1007911. doi: 10.1371/journal.pgen.1007911 (PMC6345494; doi:10.1371/journal.pgen.1007911)

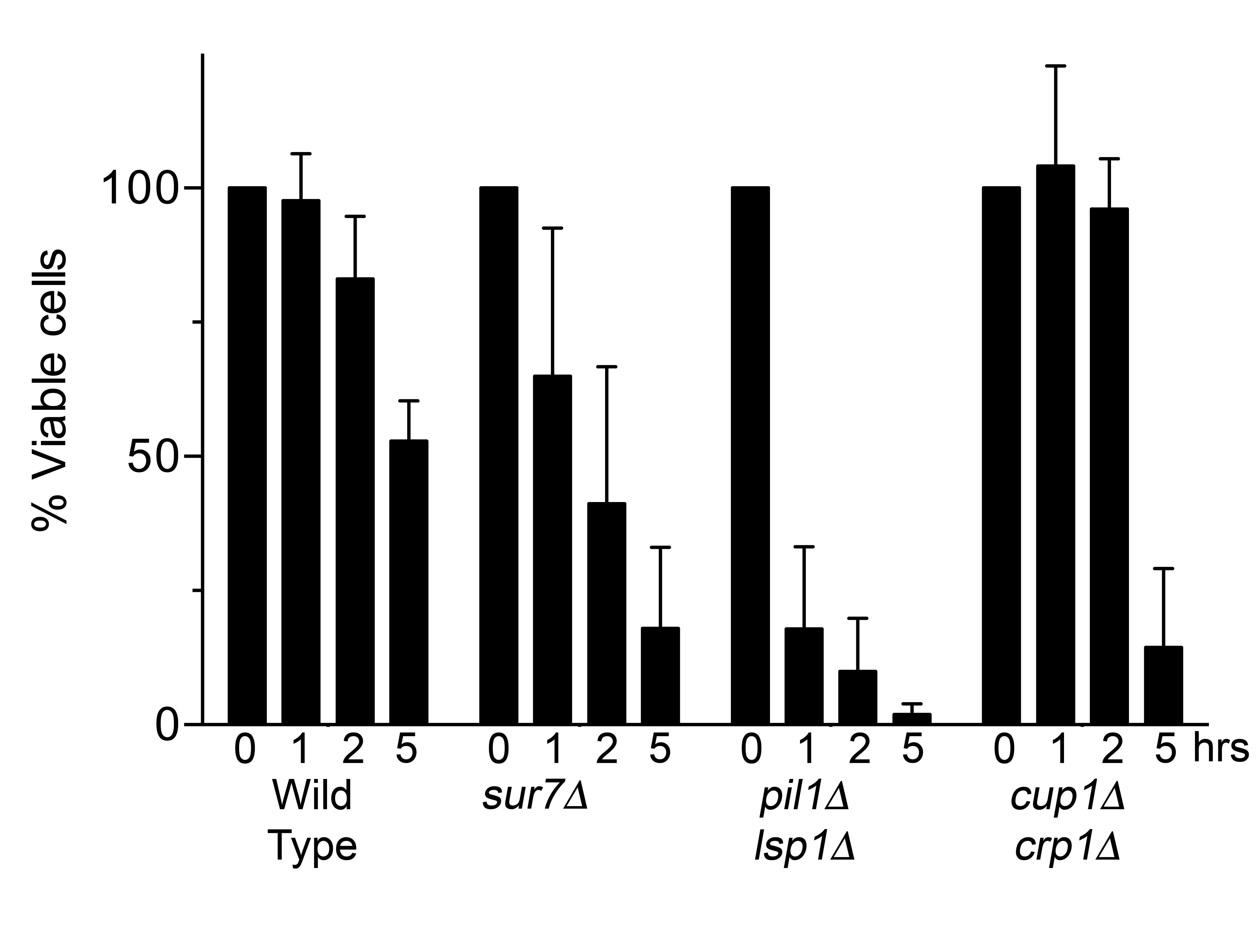

Supplement: S1 Fig — C. albicans strains were incubated in a solution of CuSO4 for the indicated time, and then the viability of the cells was determined by plating cells on YPD medium and counting the CFUs. This assay was similar to that shown in Fig 1D, except that HCl was used to lower the pH. The results represent the average of three independent experiments performed on different days. Error bars indicate SD. Strains used were: WT, wild-type DIC185; sur7Δ (YJA11); sur7Δ comp. (YJA12); pil1Δ lsp1Δ (YHXW21-1); pil1Δ lsp1Δ comp (+ LSP1, YHXW23-1); and cup1Δ crp1Δ (KC25). (TIF) [file pgen.1007911.s001.tif]

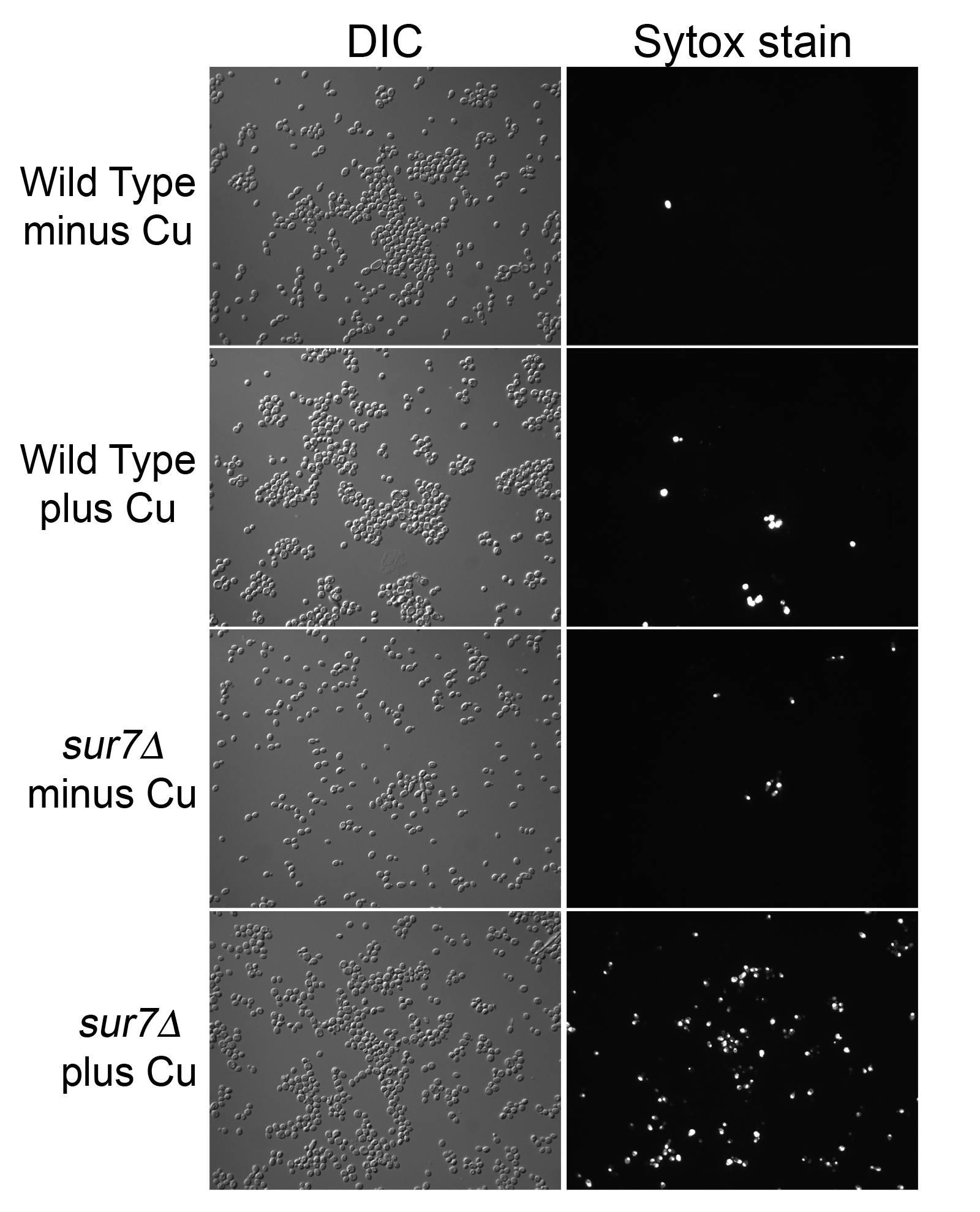

Supplement: S2 Fig — Representative samples of cells stained with the membrane impermeable dye SYTOX Green. Wild type and sur7Δ cells were incubated in the presence of 10 μM CuSO4 with 1 mM MES buffer at pH 6 for two hr. Cells were then washed, stained with SYTOX Green for 5 min, and then viewed by fluorescence microscopy. WT, wild-type DIC185; sur7Δ (YJA11). (TIF) [file pgen.1007911.s002.tif]

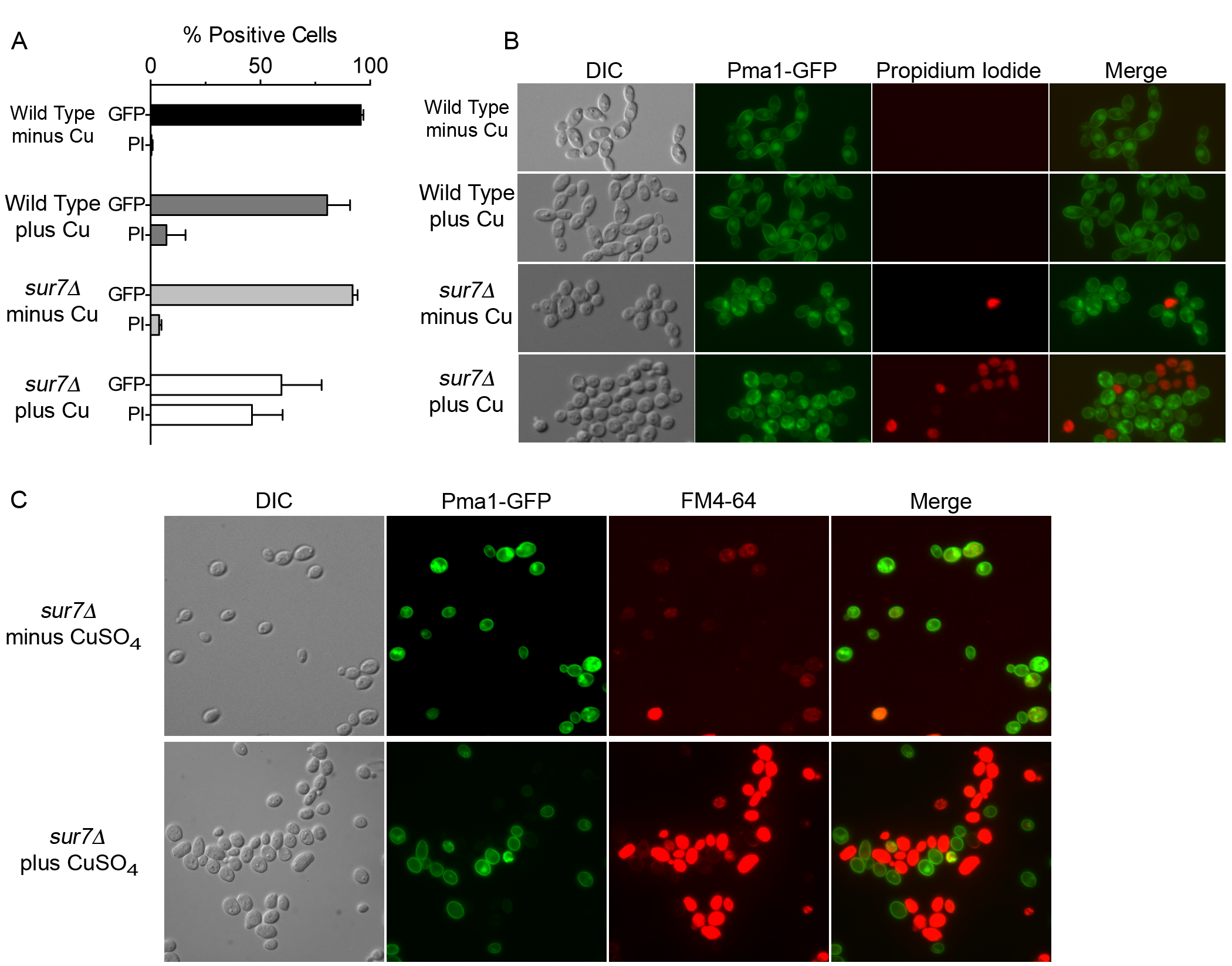

Supplement: S3 Fig — (A) Log phase sur7Δ cells engineered to produce a fusion between the plasma membrane H+ ATPase Pma1 and GFP were incubated in water or 10 μM CuSO4 for 2 hr at 37°C. Cells were then stained with the membrane-impermeable dye propidium iodide (PI). The graph depicts how copper treatment causes a loss in GFP fluorescence and an increase in membrane permeability, indicated by PI staining. (B) Photographs showing that sur7Δ PMA1-GFP cells that lost the GFP signal with CuSO4 treatment stained with PI. The graph represents averages of three independent experiments performed on different days. Strains used were the wild type control PMA1- GFPγ (YHXW11) and sur7Δ PMA1-GFPγ (YHXW61). (C) The sur7Δ strain PMA1-GFP (YHXW61) was incubated in the presence or absence of 10 μM CuSO4 with 1 mM MES buffer at pH 6 for two hr, stained with FM4-64, and then imaged by fluorescence microscopy. Note that loss of Pma1-GFP correlated with intense staining by FM4-64. (TIF) [file pgen.1007911.s003.tif]

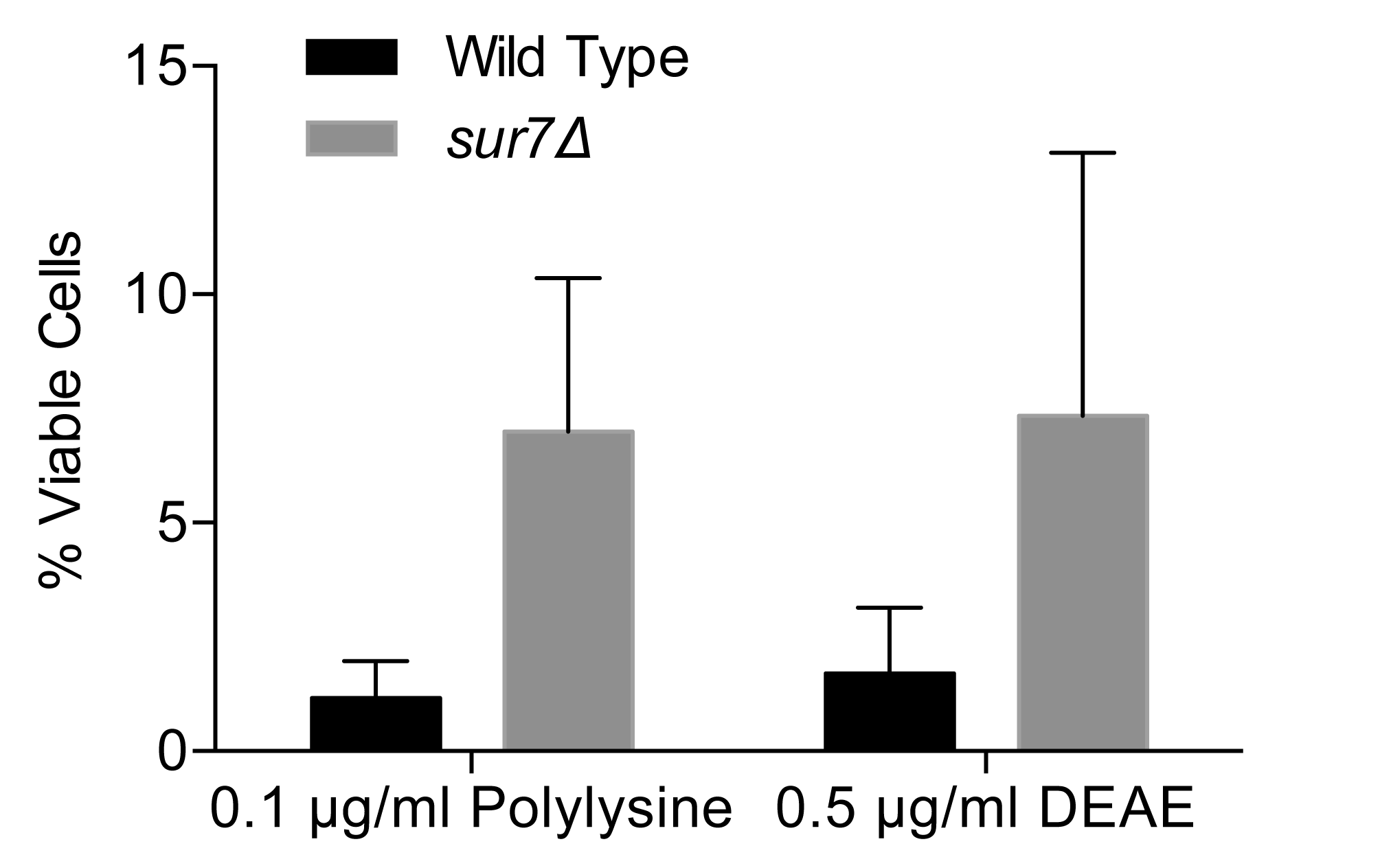

Supplement: S4 Fig — The indicated strains were incubated with DEAE dextran hydrochloride (500 kDa) or poly-L-lysine hydrobromide (30 kDa) for 2 hr at 37°C. Samples were then plated onto YPD medium, incubated at 30°C for 48 hr, and then CFUs were counted to assess viability. WT, wild-type DIC185; sur7Δ (YJA11). (TIF) [file pgen.1007911.s004.tif]

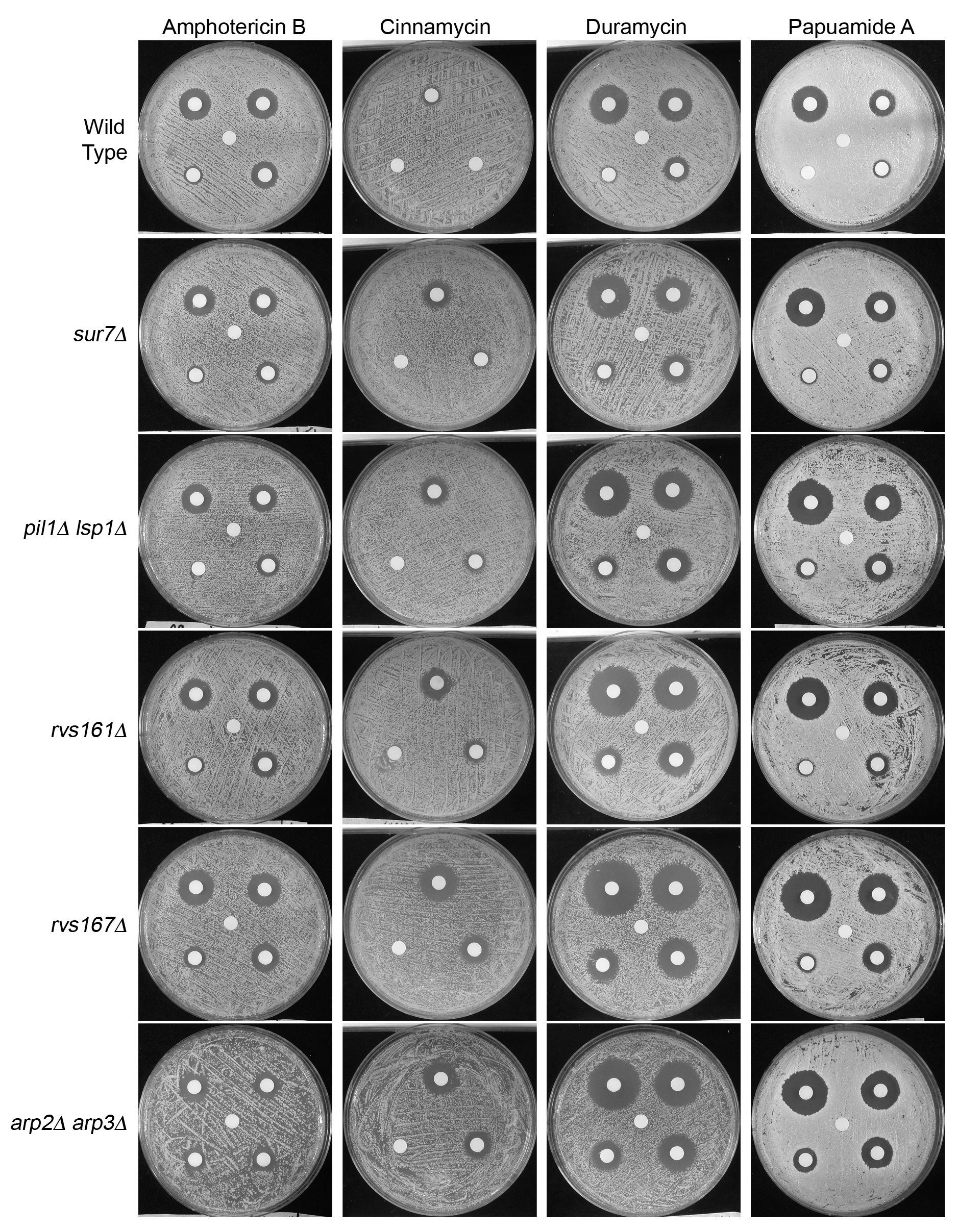

Supplement: S5 Fig — Representative halo assay for testing the sensitivity of cells to different drugs. A lawn of 2.5 x 10^5^ cells was spread on the surface of a synthetic medium agar plate, and then paper filter disks containing 10 μl of different concentrations of the drug were applied to the surface of the plate. After incubation for 48 hr at 30°C, the plates were photographed. Concentrations used for amphotericin were 500, 250, 125, 50, and 0 μg/ml. Concentrations used for cinnamycin were 40, 20, and 0 μg/ml. Concentrations used for duramycin were 20, 10, 5, and 2.5 μg/ml and 0 μg/ml. Concentrations used for papuamide A were 1000, 500, 250, 125, and 0 μg/ml. Strains used were DIC185, sur7Δ (YJA11), rvs161Δ (YLD14-3), rvs167Δ (YLD16), and arp2Δ arp3Δ (CaEE27) (TIF) [file pgen.1007911.s005.tif]
