# Supplementary material for: Plasma membrane architecture protects Candida albicans from killing by copper
Source: PLoS Genet. 2019 Jan 11;15(1):e1007911. doi: 10.1371/journal.pgen.1007911 (PMC6345494; doi:10.1371/journal.pgen.1007911)
Supplement: S1 Table — (DOCX) [file pgen.1007911.s006.docx]

**Supporting Information Table. Fatty Acid Analysis**

| Expt. 1 |  |  |  |  | Expt. 2 |  |  |  |  |  |
| --- | --- | --- | --- | --- | --- | --- | --- | --- | --- | --- |
|  | **% of Total Fatty Acid** | | |  |  | **% of Total Fatty Acid** | | | | |
| Fatty Acid | WT | *sur7Δ* | *sur7Δ* Compl. |  | Fatty Acid | WT | *sur7Δ* | sur7 Compl | *pil1Δ* *lsp1Δ* | *pil1Δ* *lsp1Δ* Compl. |
| 14:00 | 0.39 | 0.46 | 0.43 |  | 14:00 | 0.73 | 1.17 | 0.95 | 1.55 | 1.06 |
| 15:00 | 0.18 | 0.2 | 0.21 |  | 15:00 | 0.3 | 0.7 | 0.35 | 0.75 | 0.38 |
| 16:00 | 11.04 | 11.7 | 10.69 |  | 16:00 | 15.13 | 15.85 | 15.56 | 17.49 | 15.2 |
| 16:1 w7c^1^ | 10.33 | 10.84 | 11.32 |  | 16:1 w7c^1^ | 9.18 | 7.82 | 10.16 | 9.22 | 9.95 |
| 17:00 | 0.08 | 0.1 | ND^5^ |  | 17:00 | 0.43 | 0.57 | 0.46 | 0.55 | 0.37 |
| 17:1 w8c^2^ | 0.38 | 0.37 | 0.39 |  | 17:1 w8c^2^ | 0.99 | 1.26 | 1.22 | 1.41 | 1.01 |
| 18:00 | 3.88 | 3.77 | 3 |  | 18:00 | 4.16 | 6.28 | 3.5 | 4.75 | 3.6 |
| 18:1 w8c^2^ | 12.53 | 11.16 | 14.67 |  | 18:1 w8c^2^ + w9c^3^ | 42.13 | 36.42 | 40.35 | 36.08 | 42.3 |
| 18:1 w9c^3^ | 30.78 | 32.07 | 27.42 |  |  |  |  |  |  |  |
| 18:2 w6c^4^ | 27.58 | 26.55 | 28.9 |  | 18:2 w6c^4^ | 25.52 | 26.33 | 25.07 | 25.17 | 23.93 |

^1^ w7c indicates unsaturated bond is at the omega 7 position and in a cis configuration; *cis-Δ^9^*.

^2^ w8c indicates unsaturated bond is at the omega 8 position and in a cis configuration; *cis-Δ^9^*.

^3^ w9c indicates unsaturated bond is at the omega 9 position and in a cis configuration; *cis-Δ^9^*.

^4^ w6c indicates unsaturated bond is at the omega 6 position and in a cis configuration; *cis,cis-Δ^9^,Δ^12^*.

^5^ ND indicates Not Detected.
